# Superresolution microscopy reveals linkages between ribosomal DNA on heterologous chromosomes

**DOI:** 10.1083/jcb.201810166

**Published:** 2019-07-03

**Authors:** Tamara A. Potapova, Jay R. Unruh, Zulin Yu, Giulia Rancati, Hua Li, Martha R. Stampfer, Jennifer L. Gerton

**Affiliations:** 1 Stowers Institute for Medical Research, Kansas City, MO; 2 Institute of Medical Biology, Agency for Science, Technology and Research, Singapore; 3 Biological Systems and Engineering Division, Lawrence Berkeley National Laboratory, Berkeley, CA; 4 Department of Biochemistry and Molecular Biology, University of Kansas Medical Center, Kansas City, KS

## Abstract

The spatial organization of the genome is enigmatic. Direct evidence of physical contacts between chromosomes and their visualization at nanoscale resolution has been limited. We used superresolution microscopy to demonstrate that ribosomal DNA (rDNA) can form linkages between chromosomes. We observed rDNA linkages in many different human cell types and demonstrated their resolution in anaphase. rDNA linkages are coated by the transcription factor UBF and their formation depends on UBF, indicating that they regularly occur between transcriptionally active loci. Overexpression of c-Myc increases rDNA transcription and the frequency of rDNA linkages, further suggesting that their formation depends on active transcription. Linkages persist in the absence of cohesion, but inhibition of topoisomerase II prevents their resolution in anaphase. We propose that linkages are topological intertwines occurring between transcriptionally active rDNA loci spatially colocated in the same nucleolar compartment. Our findings suggest that active DNA loci engage in physical interchromosomal connections that are an integral and pervasive feature of genome organization.

## Abstract (precis)

Potapova et al. use superresolution microscopy to describe linkages between ribosomal DNA on heterologous human chromosomes whose formation depends on the transcription factor UBF and topoisomerase II. Linkages persist in the absence of cohesion but require topoisomerase II for resolution.

## Introduction

The human genome is packaged into 23 pairs of chromosomes that occupy distinct territories in the nucleus and can form interchromosomal contacts (Cremer et al., 1993; Bolzer et al., 2005; Meaburn and Misteli, 2007). Contacts between chromosomes may provide a structural basis for long-range genetic interactions (Spilianakis and Flavell, 2004; Williams et al., 2010; Kim et al., 2014). A recent study using labeling of specific genomic locations by CRISPR/Cas9 in combination with Hi-C concluded that interchromosomal interactions may be as common as intrachromosomal interactions (Maass et al., 2018). While interchromosomal associations exist in interphase nuclei, the nature of these associations is unknown, as is the mode of resolution during mitosis.

**Figure 1.**
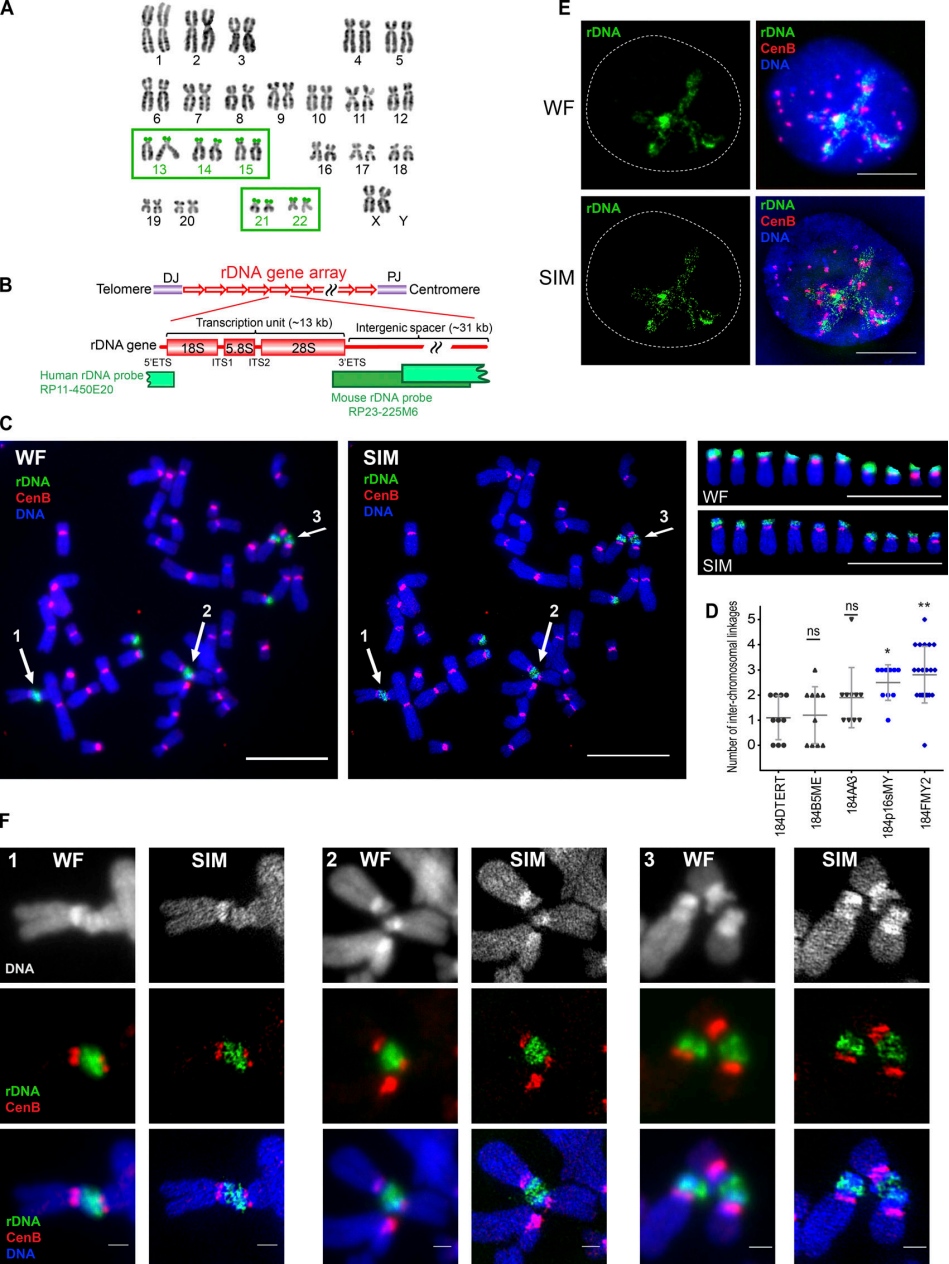
**SIM revealed rDNA linkages between acrocentric human chromosomes. (A)** Normal human karyotype with highlighted acrocentric chromosomes 13, 14, 15, 21, and 22 bearing rDNA loci on their short arms, provided courtesy of Karen Miga (Genomics Institute, University of California Santa Cruz, Santa Cruz, CA) and Amalia Dutra (Cytogenetic and Microscopy Core, National Human Genome Research Institute, National Institutes of Health, Bethesda, MD). **(B)** Schematic representation of rDNA repeat units and coverage of rDNA probes used in this study. In the human karyotype, rDNA genes are arranged as repeats on the short arms of the acrocentric chromosomes between centromeres and telomeres, flanked by proximal and distal junctions (PJ and DJ). Each unit consists of a coding region (encoding pre-mRNA for 18S, 5.8S, and 28S ribosomal RNA subunits) and intergenic spacer. Boundaries of the coding region contain external transcribed spacers (5′ETS and 3′ETS), and coding parts of the 45S sequence are separated by internal transcribed spacers (ITS1 and ITS2). The human rDNA probe used in this study was derived from BAC clone RP11-450E20 and spans the intergenic spacer and the transcription initiation site of the next repeat. The mouse rDNA probe was derived from BAC clone RP23-225M6, spanning the end of the coding part and the intergenic spacer. **(C)** Wide-field illumination (WF) and SIM images of mitotic chromosome spread from 184FMY2 HMEC cell labeled by FISH with rDNA probe (green) and CenB probe (red). Arrows 1–3 point to acrocentric chromosomal rDNA associations. Panels on the right show individual acrocentric chromosomes: six large rDNA chromosomes and four small rDNA chromosomes. Bar, 10 µm. **(D)** Quantification of the number of interchromosomal rDNA linkages in chromosomal spreads from isogenic HMEC cell lines labeled by FISH with rDNA probe. Cell lines overexpressing c-Myc highlighted in blue. Images of ≥10 chromosomal spreads from each cell line were examined. The difference between 184DTERT and each of the other cell lines was evaluated using the Mann–Whitney *U* test. *, P < 0.05; **, P < 0.001; ns, not significant. **(E)** Wide-field illumination and SIM images of the interphase nucleus of 184FMY2 HMEC cell labeled by FISH with rDNA probe (green) and centromere CenB probe (red). While centromere loci form compact dots, most of the rDNA forms long thin filaments within the nucleolar compartment. Bar, 10 µm. **(F)** Panels 1–3 show corresponding enlarged wide-field illumination and SIM images of rDNA associations marked by arrows in C. While wide-field illumination images accurately depict rDNA associations, SIM images reveal the network of thin filamentous rDNA linkages between different acrocentric chromosomes. Bar, 1 µm.

The 45S ribosomal DNA (rDNA) genes present a potential paradigm of interchromosomal interactions because they are present on multiple chromosomes that regularly associate together in interphase nuclei to form the nucleolus (McStay, 2016). 45S genes in the human genome are present in several hundred nearly identical copies (Scherer, 2008); their actual number varies among individuals (Gibbons et al., 2015; Xu et al., 2017a). They are organized in tandem repeats that are partitioned among the short arms of five acrocentric chromosome pairs: 13, 14, 15, 21, and 22 (Henderson et al., 1972; Fig. 1 A). Each rDNA repeat unit consists of the coding region producing the transcript to be processed into 28S, 5.8S, and 18S rRNAs and a long noncoding intergenic spacer (Fig. 1 B).

Pioneering cytogenetic studies of human chromosomal spreads from peripheral blood samples demonstrated “satellite associations” between the short arms of acrocentric chromosomes (Ferguson-Smith and Handmaker, 1961; Zhdanova, 1972; Jacobs et al., 1976; Ardito et al., 1978). These barely visible connections were distinct from Robertsonian translocations because chromosomes clearly remained separated, could be positioned at an angle, and occasionally formed triple and even quadruple associations, described as “acrobats holding hands” (Ferguson-Smith and Handmaker, 1961). Subsequent studies identified changes in the number of satellite associations under many different pathological conditions (Frolov et al., 1975; Hansson, 1979; Lezhava, 1979; Yasseen and Al-Musawi, 2001; Caradonna, 2015). That said, the presence of acrocentric associations per se did not seem to be pathological, because they were always present in controls too. Moreover, acrocentric associations in chromosomal spreads from chimpanzee leukocytes were also observed (Ardito et al., 1973). Despite these exciting early findings, the physical nature of these associations has remained entirely unknown.

We used structural illumination superresolution microscopy (SIM; Gustafsson, 2000; Heintzmann and Huser, 2017) to demonstrate that rDNA can form inter- and intrachromosomal connections, or linkages. We speculate that they form in the nucleolus during interphase as a consequence of rDNA transcription. We suggest that rDNA linkages may be DNA catenations because their formation and resolution depend on the activity of topoisomerase II. Our findings indicate that active transcription can generate physical interchromosomal connections at specific genomic regions. These connections are a reproducible and modulable feature of genome organization.

## Results

### Linkages between rDNA regions of acrocentric chromosomes are ubiquitous in human cells

We used conventional microscopy and SIM to study chromatin organization of the rDNA. rDNA repeats were directly labeled by FISH using rDNA probes labeled with fluorescent dyes. In the course of this study, we examined rDNA in multiple human, mouse, and human–mouse hybrid cell lines. The human rDNA probe was generated from a BAC clone spanning the rDNA repeat sequence from intergenic spacer to the origin of transcription of the next repeat unit. The mouse rDNA probe spanned the end of the coding region and a large part of the intergenic spacer (Fig. 1 B).

Initially, we examined a panel of isogenic human mammary epithelial cells (HMECs) immortalized following exposure to a chemical carcinogen and/or by transduction of oncogenes, or by expression of human telomerase (Stampfer and Bartley, 1985; Stampfer et al., 2001, 2003; Garbe et al., 2014). Chromosome spreads from HMECs imaged by wide-field microscopy revealed physical connections between acrocentric chromosomes at the rDNA loci (Fig. 1 C, left panel, arrows 1–3). The frequency of these connections was particularly high in the HMEC line 184FMY2 (Fig. 1 D) that was immortalized following transduction of normal HMECs with c-Myc (Garbe et al., 2014; Lee et al., 2015). Superresolution imaging revealed that these associations are composed of thin threadlike filaments (Fig. 1 C, middle panel, arrows 1–3). Panels on the right show wide-field and SIM images of the 10 individual acrocentric chromosomes present in this chromosomal spread. In interphase nuclei from 184FMY2 HMECs, most of the rDNA was decondensed, extending continuous long fibers that organize the nucleolus (Fig. 1 E).

A magnified view of individual linkages (Fig. 1 F) shows that rDNA connections were not chromosome fusions or Robertsonian translocations: first, rDNA chromosomes often appeared to be joined at an angle or laterally, and second, these connections were not stained prominently with classic fluorescent DNA dyes such as DAPI or Hoechst 33342. Linkages were only unambiguously detected by rDNA FISH labeling. While wide-field images accurately detected rDNA associations, SIM images revealed a network of fine filaments between sister chromatids (intrachromosomal linkages) and between different acrocentric chromosomes (interchromosomal linkages).

To ensure that linkages were not reconstruction artifacts of SIM data, several quality control measures were undertaken. First, the data quality was evaluated using SIMcheck (Ball et al., 2015). Imaging data passed all listed criteria such as modulation contrast-to-noise ratio, reconstructed Fourier plot, channel intensity variation, spherical aberration mismatch, and reconstructed intensity histogram (Fig. S1 A). Second, the fidelity of image reconstruction was tested by imaging chromosomes hybridized to the rDNA probe labeled with two fluorophores with nonoverlapping excitation and emission properties: fluorescein (green) and ROX (red). Reconstructed SIM images of green and red labels showed the same structures in both channels (Fig. S1 B). Spatial Pearson cross-correlation analysis of green and red images indicated strong spatially dependent colocalization (Fig. S1 C). Third, rDNA linkages were imaged using an independent superresolution technique, stimulated emission depletion (STED) microscopy (Hell and Wichmann, 1994; Müller et al., 2012). STED imaging showed linkages similar to those shown by SIM (Fig. S1 D).

**Figure 2.**
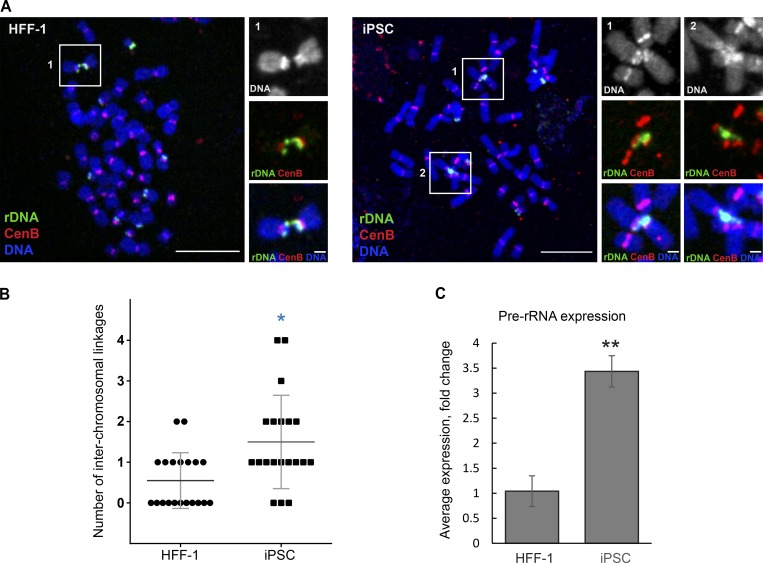
**Frequency of rDNA linkages and rRNA synthesis in HFF-1 and derivative iPS cells. (A)** Confocal images of mitotic chromosome spread from HFF1 (left) and iPSC (right) cell labeled by FISH with rDNA probe (green) and CenB probe (red). Bar, 10 µm. Magnified insets depict acrocentric chromosomal associations between rDNA (bar, 1 µm). **(B)** Quantification of the number of interchromosomal rDNA linkages in chromosomal spreads from HFF-1 and iPSC labeled by FISH with rDNA probe. High-resolution confocal images of 20 chromosomal spreads from each cell line were examined. The Mann–Whitney *U* test was used to compare iPSC with parental HFF-1. *, P < 0.05. **(C)** Real-time qPCR analysis of iPSC compared with the parental HFF-1 cell line. The expression of pre-rRNA was normalized to the expression of GAPDH mRNA. Bar heights represent an average fold change of three primer sets to the 5′ETS; error bars represent SD. Statistical significance was evaluated using *t* test; **, P < 0.001.

Interchromosomal linkages can connect large and small acrocentric chromosomes that are obviously heterologous. Most frequently, linkages were formed between two chromosomes, but they occasionally connected three chromosomes or more. Further examination of multiple different human cell lines (RPE1, chondrocyte [CHON-002], HCT116, LoVo, NCI-H209, and primary human lymphocytes) showed that rDNA linkages were present in chromosomal spreads from all cell cultures, and any rDNA chromosome can be connected to any other. Surveying rDNA linkages in normal human cell lines, we examined primary human foreskin fibroblast cell line (HFF-1) and induced pluripotent stem cells (iPSCs) DYS0100 that were derived from HFF-1 (Wren et al., 2015). Human iPS cells displayed interchromosomal rDNA connections with higher frequency than their precursor HFF1 fibroblasts (Fig. 2, A and B). Stem cells are known to have a high rate of rRNA synthesis and ribosomal biogenesis (Watanabe-Susaki et al., 2014). We measured levels of nascent rRNA transcripts by quantitative PCR (qPCR), using three primer sets to the 5′ externally transcribed spacer (5′ETS) region of the pre-rRNA that is not retained after rRNA processing. The higher number of rDNA linkages in iPSC positively correlated with the increased levels of nascent transcription (Fig. 2 C). Together with previous reports of acrocentric associations, our data indicate that linkages between rDNA loci may be a ubiquitous feature of human chromosomes.

In the human genome, 45S genes are positioned on the short arms of acrocentric chromosomes between centromeres and telomeres, separated from telomeres by only a relatively short sequence called the distal junction (Fig. 1 B; Floutsakou et al., 2013). Immortal cell lines in culture often have short telomeres that may be difficult to detect by FISH. Expression of a dominant-negative mutant allele of the telomere protein TRF2 in human fibrosarcoma HT1080 cell line caused an increase in a number of rDNA connections, interpreted by the authors as prefusogenic (Stimpson et al., 2014). To examine telomere integrity in the context of rDNA linkages, we obtained chromosomal spreads from primary human male lymphocytes and labeled them by FISH with rDNA and telomere probes. rDNA linkages in primary lymphocytes were not very frequent, but they clearly coexisted with intact telomeres, indicating that rDNA linkages were not telomere fusions (Fig. S2 A). Moreover, RNase A did not destroy rDNA linkages (Fig. S2 B), indicating that linked loci are not held together by RNA.

In contrast to human chromosomes, mouse chromosomes are telocentric and mouse rDNA loci are located distally from centromeres, isolated from telomeres by centromeric heterochromatin (Cazaux et al., 2011). In chromosomal spreads from mouse cell lines, rDNA linkages were infrequent. However, we observed rDNA linkages in chromosomal spreads from mouse stem cells (Fig. S2 C). Like human linkages, mouse linkages consisted of thin threads that were not labeled with DAPI. The “telomere-centromere-rDNA” arrangement of mouse chromosomes means rDNA linkages cannot be telomere fusions.

### c-Myc promotes formation of rDNA linkages

**Figure 3.**
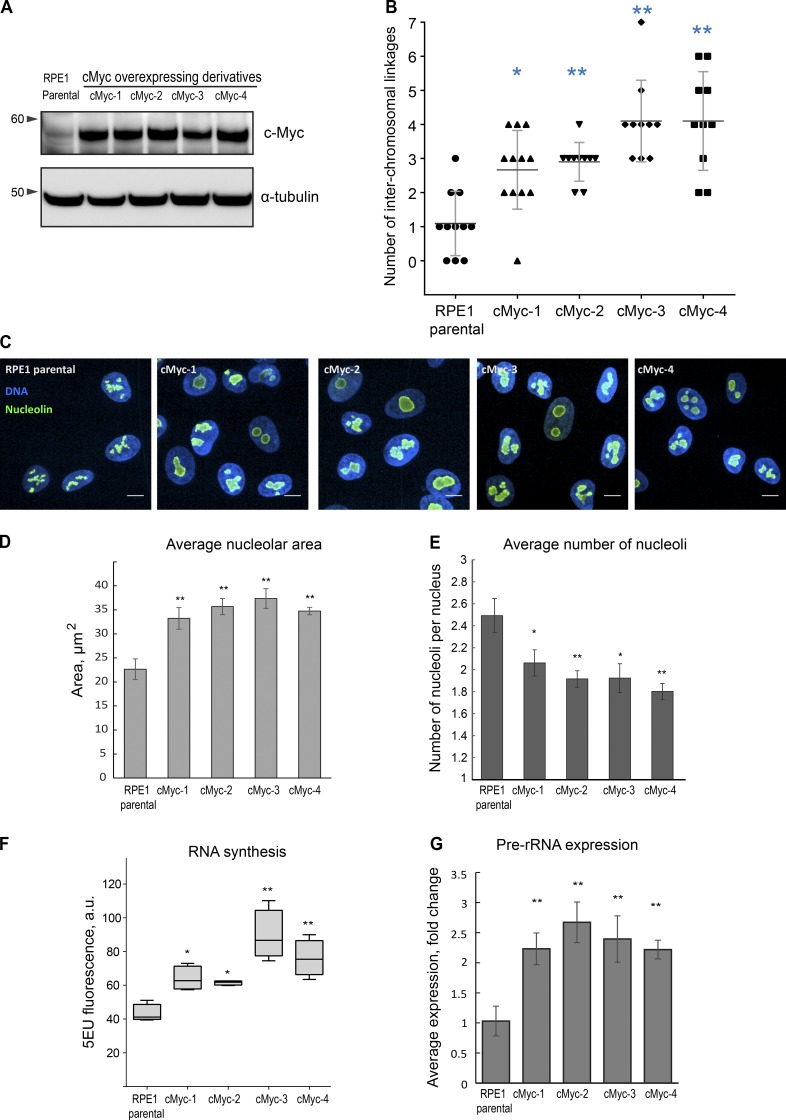
**Overexpression of c-Myc leads to an elevated number of rDNA linkages, increased nucleolar size and merging, and a higher level of rRNA synthesis. (A)** Western blot analysis of c-Myc protein levels of parental RPE1 cell line and c-Myc–overexpressing single-cell clone derivatives cMyc-1, cMyc-2, cMyc-3, and cMyc-4. **(B)** Quantification of the number of interchromosomal rDNA linkages in chromosomal spreads from parental RPE1 cells and c-Myc–overexpressing derivatives labeled by FISH with rDNA probe. High-resolution confocal images of ≥10 chromosomal spreads from each cell line were examined. The Mann–Whitney *U* test was used to compare c-Myc–overexpressing samples with parental RPE1. *, P < 0.05; **, P < 0.001. **(C)** Representative spinning disk confocal images of nucleolin immunofluorescence (green) of parental RPE1 cells and c-Myc–overexpressing derivatives. Nuclei were counterstained with DAPI (blue). Note enlarged and merged nucleoli. Bar, 10 µm. **(D and E)** Quantification of nucleolar area (D) and number (E) based on the nucleolin immunofluorescence labeling as in C show enlargement of the nucleoli and their decreased number in c-Myc–overexpressing cells. Bars show averages of three large fields of view (montage images) containing tens to hundreds of cells. *, P < 0.05; **, P < 0.001. Statistical significance was evaluated using one-way ANOVA with Dunnett multiple comparisons, comparing c-Myc samples to parental control. Error bars denote SD. **(F)** 5-EU incorporation in parental RPE1 cells and c-Myc–overexpressing single-cell clone derivatives. 5-EU–labeled RNA was detected with fluorescent azide and quantified by high-throughput imaging. Statistical significance was evaluated using one-way ANOVA with Dunnett multiple comparisons. *, P < 0.05; **, P < 0.001. **(G)** Real-time qPCR analysis of pre-rRNA expression of c-Myc–overexpressing derivatives compared with the parental RPE1 cells. The expression of pre-rRNA was normalized to the expression of GAPDH mRNA. Bar heights represent an average fold change of three primer sets to the 5′ETS; error bars represent SD. Statistical significance was evaluated using *t* tests; **, P < 0.001.

c-Myc regulates several key steps in ribosomal biogenesis, including transcription and processing of ribosomal RNA (van Riggelen et al., 2010). c-Myc binds to rDNA directly and accelerates transcription of 45S genes (Grandori et al., 2005). The increased incidence of linkages in 184FMY2 prompted us to investigate the role of c-Myc in linkage formation. For this, we transduced the human hTERT-immortalized RPE1 cell line with c-Myc containing lentivirus and selected four isogenic single-cell clones overexpressing c-Myc, designated cMyc 1–4 (Fig. 3 A).

The parental RPE1 cell line has 46–47 chromosomes, with nine chromosomes bearing 45S genes in most cells (Fig. S3, A and B). In this cell line, one rDNA locus on one of the small acrocentric chromosomes (likely chromosome 22) is absent. Some RPE1 cells contained 47 chromosomes because they tend to gain an extra copy of chromosome 12 with passages (Potapova et al., 2016). c-Myc–overexpressing RPE1 derivatives stably maintain a diploid karyotype, with the same number of rDNA chromosomes (Fig. S3 A). One of the derivatives (cMyc-3) lost a copy of chromosome X (Fig. S3 B), which is a frequent karyotypic alteration in cell culture (Xu et al., 2017b).

Linkages were increased in all four c-Myc–overexpressing clones (Fig. 3 B), while repeat copy number was not significantly changed (Fig. S3 C). Immunofluorescence labeling for one of the key nucleolar components, nucleolin (Tajrishi et al., 2011), showed that c-Myc–overexpressing cells exhibited prominent nucleoli (Fig. 3 C). Nucleoli in c-Myc RPE1 derivatives were larger than in the parental RPE1 (Fig. 3 D), but their number was reduced (Fig. 3 E). Although the distribution of the chromatin-associated Ki67 proliferation marker reflects the enlarged nucleolar size in c-Myc derivatives, its average intensity in the nucleus was in the same range as in the parental cell line (Fig. S3 D).

Next, we measured the effect of c-Myc overexpression on rRNA synthesis by incorporation of 5-ethynyluridine (5-EU) into nascent RNA (Jao and Salic, 2008). Ribosomal RNA can account for >80% of the total cellular RNA (Palazzo and Lee, 2015); therefore, this method reflects the rate of production of ribosomal RNA. All c-Myc–overexpressing RPE1 derivatives demonstrated increased rRNA transcription (Fig. 3 F). To further validate the increased rRNA transcription, we measured the expression of preprocessed rRNA by qPCR. These measurements confirmed that the c-Myc–overexpressing cells had significantly higher levels of nascent rRNA synthesis than the parental RPE1 cell line (Fig. 3 G). Therefore, c-Myc overexpression leads to increased nucleolar size and coalescence, high level of rRNA transcription, and frequent linkages between rDNA chromosomes. Overall, c-Myc–overexpressing cells as well as the iPSCs demonstrate a positive correlation between an increased rate of rDNA transcription and an elevated frequency of rDNA linkages.

### The rDNA transcription factor upstream binding factor (UBF) is required for the formation of rDNA linkages

**Figure 4.**
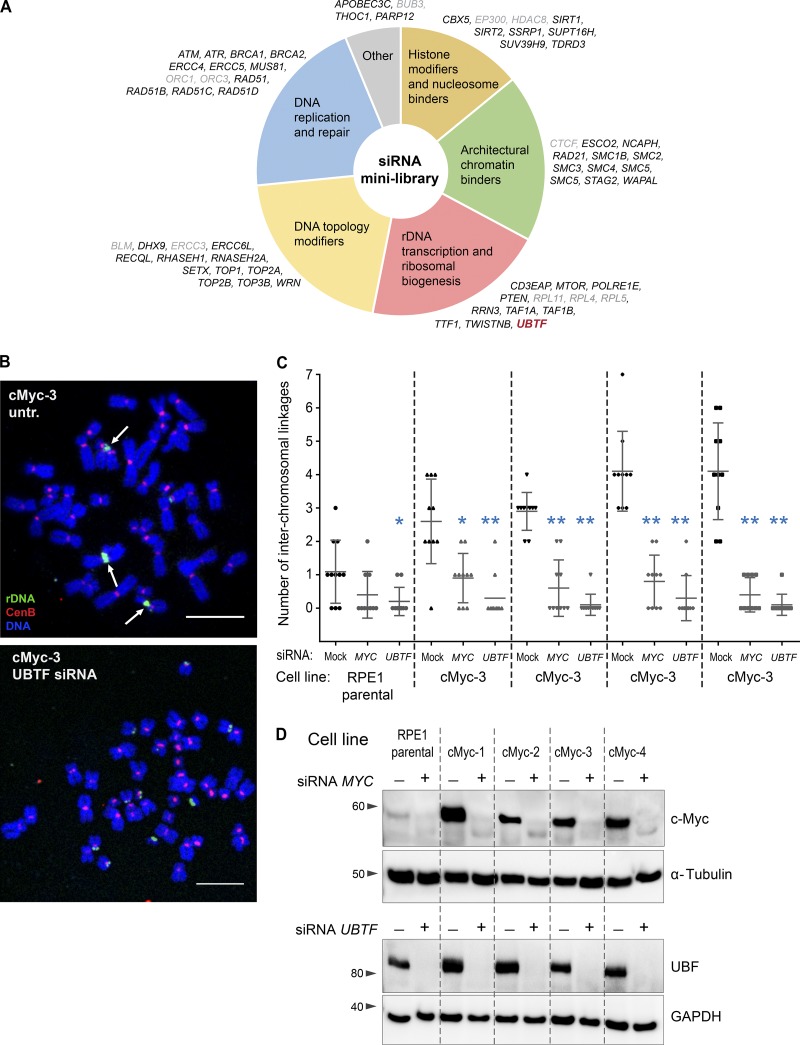
**siRNA minilibrary screen identified *UBTF* as an essential gene for the formation of rDNA linkages. (A)** Schematic representation of the siRNA minilibrary used to screen for genes that eliminate rDNA linkages in c-Myc–overexpressing cells. c-Myc–overexpressing RPE1 derivative cMyc-3 cells were transfected with siRNAs for 72 h, followed by mitotic spread preparation and FISH labeling for the rDNA. The number of interchromosomal linkages was scored manually. Genes whose knockdown prevented mitotic entry, precluding chromosomal spread preparation, are highlighted in gray. The sole hit, *UBTF*, is highlighted in red. **(B)** Representative chromosomal spreads from c-Myc–overexpressing cells and untransfected (untr.) control or transfected with *UBTF* siRNAs. Arrows point to interchromosomal linkages in the control spread. In the UBTF knockdown spread, rDNA linkages are absent. Bar, 10 µm. **(C)** Quantification of the number of interchromosomal rDNA linkages from parental RPE1 cells and c-Myc–overexpressing derivative cell lines cMyc 1–4 transfected with *MYC* or *UBTF* siRNAs. Knockdown of *UBTF* causes a stronger decrease in the frequency of rDNA linkages than the knockdown of *MYC* in all derivatives. Images of ≥10 chromosomal spreads from each sample were examined. The Mann–Whitney *U* test was used to compare siRNA-treated samples with control. *, P < 0.05; **, P < 0.001. **(D)** Western blot analysis of c-Myc and UBF protein levels in cells treated with indicated siRNAs for 72 h. Both *MYC* and *UBTF* siRNAs induced strong protein knockdowns.

To search for genes influencing the formation of rDNA linkages, a small library of siRNAs was used to knock down candidate genes involved in chromatin architecture and modifications, DNA replication and repair, rDNA transcription and ribosome biogenesis, and other processes (Fig. 4 A). c-Myc–overexpressing RPE1 derivative cells (cMyc-3), which have a high frequency of rDNA linkages, were arrested in mitosis 72 h after siRNA transfection, and if the gene knockdown did not prevent mitotic entry, chromosomal spreads were collected and labeled by FISH with the rDNA probe. The only hit in this library that abolished rDNA linkages was the siRNA against the *UBTF* gene, which encodes the rDNA transcription factor UBF.

UBF is a transcription factor that binds active rDNA repeats and is necessary for rDNA transcription (Sanij et al., 2008; Sanij and Hannan, 2009). In UBF knockdown, the frequency of mitotic cells was low, indicating that cell proliferation was suppressed but not abolished. However, in most of the chromosomal spreads collected, rDNA linkages were nearly absent (Fig. 4 B). This finding was replicated in all four c-Myc–overexpressing RPE1 derivatives: UBF knockdown greatly reduced the frequency of rDNA linkages, more so than the knockdown of c-Myc (Fig. 4 C). Knockdown of UBF also abolished linkages in the parental RPE1 line. Western blot analysis confirmed the strong knockdown of both c-Myc and UBF proteins in these experiments (Fig. 4 D). Therefore, the rDNA transcription factor UBF is required for the formation of interchromosomal rDNA linkages. A previous study showed that “satellite associations” required the promoter region of the rDNA repeat, consistent with the idea that associations depend on transcriptional activity (McDowell et al., 1994). However, our efforts to test whether pharmacological inhibitors of transcription affect the frequency of rDNA linkages in mitotic chromosomal spreads failed because these drugs prevented mitotic entry.

**Figure 5.**
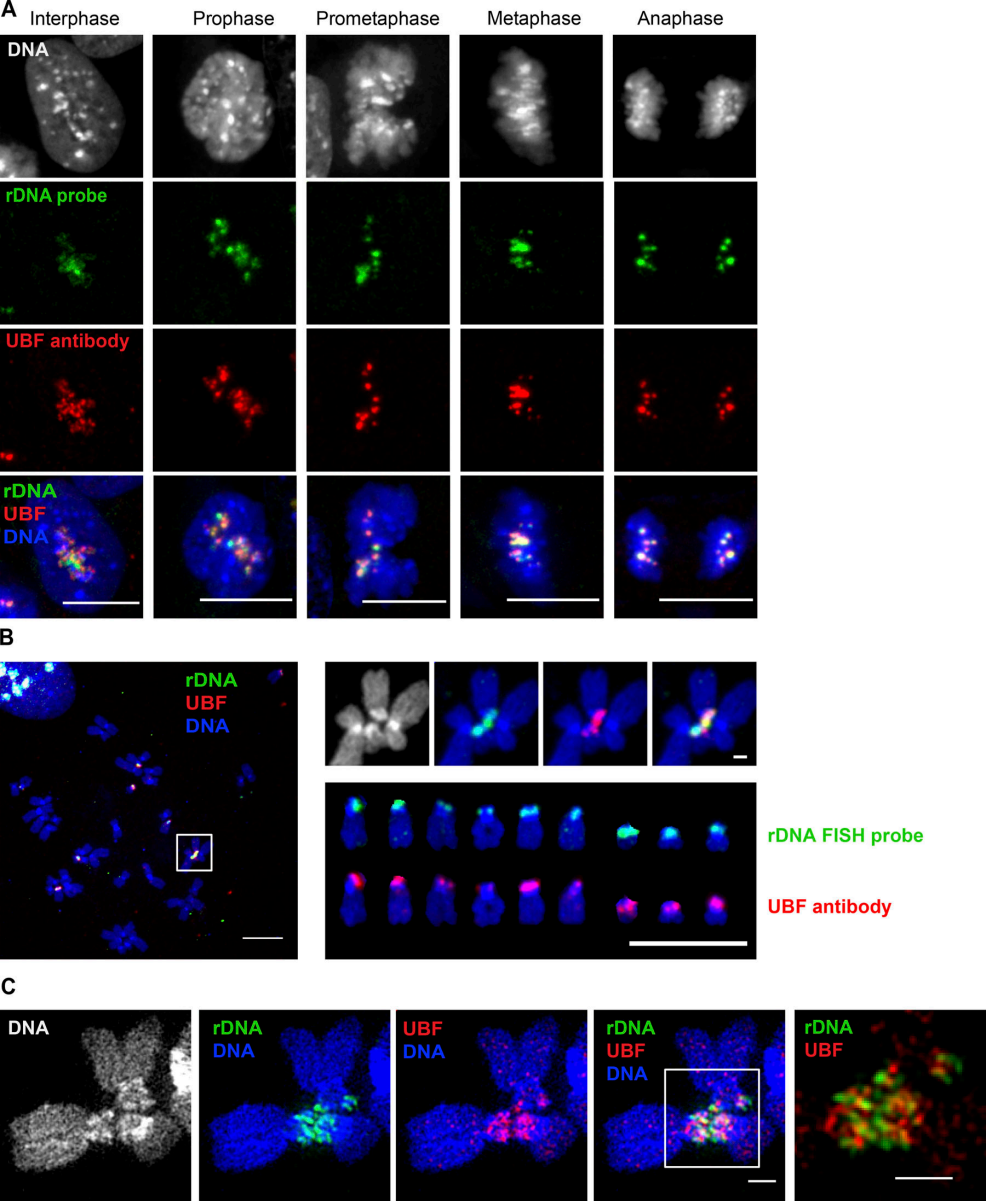
**UBF is associated with rDNA loci throughout mitotic progression and with rDNA linkages. (A)** Localization of UBF and rDNA during a progressive sequence of mitotic stages. Immuno-FISH of fixed RPE1 cells labeled with rDNA FISH probe (green) and UBF antibody (red). UBF is associated with rDNA in interphase and throughout mitotic progression. Bar, 10 µm. **(B)** A chromosomal spread from an RPE1 cell labeled by immuno-FISH with rDNA probe (green) and UBF antibody (red). The white box on the left shows the rDNA linkage shown separately on the right (top). Both rDNA and UBF form a bridge between two chromosomes. The panel on the lower right shows individual acrocentric chromosomes labeled with rDNA probe and UBF antibody, respectively. All rDNA loci in this cell line contain UBF. Bar, 10 µm. **(C)** SIM images of rDNA-linked mitotic chromosomes from c-Myc–overexpressing RPE1 cell line cMyc-3 labeled by immuno-FISH with rDNA probe (green) and UBF antibody (red). Both rDNA and UBF form filamentous connections between chromosomes. Bar, 1 µm.

UBF binds to rDNA arrays during interphase and mitosis (Roussel et al., 1993; O’Sullivan et al., 2002; Mais et al., 2005). Immuno-FISH labeling of UBF together with rDNA in mitotic RPE1 cells showed that UBF stays associated with the rDNA as it condenses and forms distinct loci during mitotic entry and segregates to two daughter cells during mitotic exit (Fig. 5 A). Immuno-FISH labeling of UBF and rDNA in RPE1 cells showed that in this cell line, all nine rDNA chromosomes are positive for UBF (Fig. 5 B). Importantly, rDNA linkages that connect acrocentric chromosomes also contain UBF (Fig. 5 B, magnified inset). Superresolution imaging showed that UBF together with rDNA forms a network of thin fibers connecting different acrocentric chromosomes (Fig. 5 C). UBF and rDNA were clearly part of the same structure, yet they did not colocalize precisely, since UBF localizes primarily to the coding part of the rDNA gene (Zentner et al., 2011; Herdman et al., 2017; Mars et al., 2018), while rDNA probes used in this study cover the noncoding part of the gene (Fig. 1 B).

### Silent rDNA loci may not form linkages

UBF marks transcriptionally active rDNA repeats and controls the number of actively transcribed ribosomal RNA genes in the cell (Sanij et al., 2008). Therefore, we asked if the UBF-dependent transcriptional activity in the nucleolar compartment was underlying the ability of rDNA to form interchromosomal linkages. We addressed this question by evaluating linkage formation between active versus silent rDNA loci present in the same cell. For this, we used two experimental systems: mouse–human hybrid cells in which human rDNA is silenced by nucleolar dominance, and tetraploid human cells in which one to six rDNA loci are silenced.

Nucleolar dominance is a well-described phenomenon observed in interspecies hybrid cells and organisms in which rDNA from only one species is transcriptionally active and rDNA from the other species is silenced (Tucker et al., 2010) We tested several somatic monochromosomal mouse–human hybrids that carried specific human rDNA chromosomes (chromosomes 21 and 22) in the mouse karyotypic background. As expected, human rDNA loci in these hybrids did not contain UBF, and rDNA linkages between human and mouse chromosomes were not observed. However, the copy number of human chromosomes in these cells was low, often one per cell, which would quantitatively limit the availability of human rDNA to contact other rDNA repeats. Because of this, we obtained a multichromosomal somatic human–mouse hybrid cell line GM15292 that contained several human rDNA chromosomes. GM15292 cell line was constructed by fusing human fibroblasts with mouse RAG cells (Vortkamp et al., 1991). This cell line is hyperploid, with chromosomal spreads containing anywhere from 70 to >100 chromosomes. While the majority of chromosomes in its karyotype are derived from mouse, it contains multiple human chromosomes including rDNA chromosomes, with the number varying from cell to cell. We made mitotic spreads from this cell line and labeled them by immuno-FISH with human and mouse rDNA probes marked with different fluorophores and UBF antibody, which recognizes both species. Human and mouse FISH probes derived from the least conserved noncoding parts of the rDNA repeat (Fig. 1 B) allowed us to distinguish mouse and human rDNA loci unambiguously.

**Figure 6.**
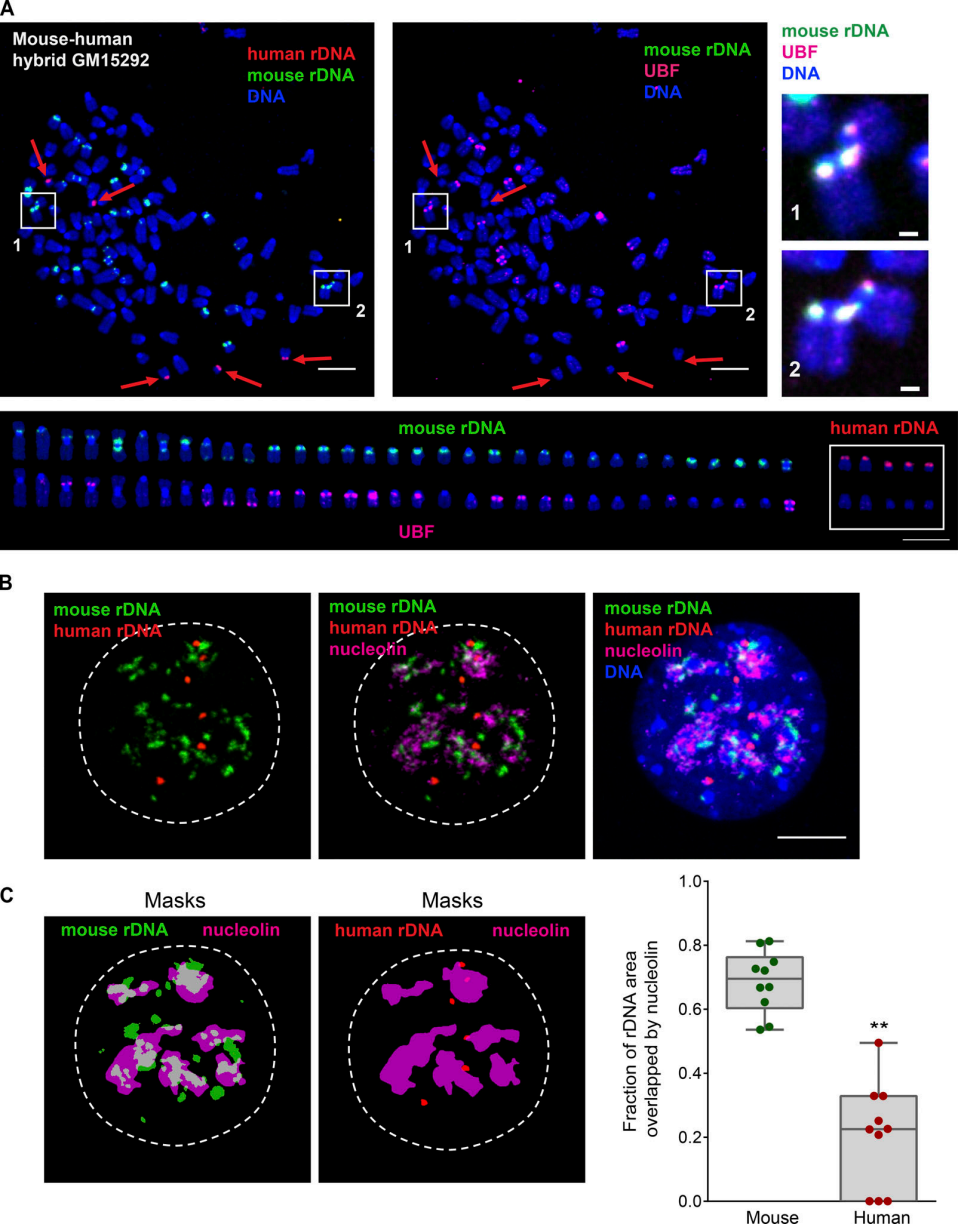
**Human–mouse hybrid cell line GM15292 displays rDNA linkages only between active (UBF^+^) mouse rDNA loci. (A)** A representative mitotic chromosome spread from mouse–human hybrid cell line GM15292 labeled by immuno-FISH with mouse rDNA probe (green), human rDNA probe (red), and UBF antibody (magenta). Boxes 1 and 2 highlight rDNA linkages (magnified on the right; bar, 1 µm). Red arrows point to human rDNA chromosomes. The lower panel shows individual mouse and human rDNA chromosomes arranged according to their species and size. The top row shows rDNA probe labeling, and the bottom row shows UBF antibody labeling of the same chromosomes. This particular spread had 112 total chromosomes, of which 33 were mouse rDNA chromosomes (19 UBF^+^) and five were human rDNA chromosomes. Note that rDNA linkages were formed only between mouse rDNA chromosomes positive for UBF (active). In all human rDNA chromosomes, loci with rDNA were UBF negative (silenced) and did not form linkages. At least 10 chromosomal spreads were examined. Bar, 10 µm. **(B)** An example of the interphase nucleus from mouse–human hybrid cell line GM15292 labeled by immuno-FISH with mouse rDNA probe (green), human rDNA probe (red), and nucleolin antibody (magenta). While most mouse rDNA is decompacted and associated with nucleolin, human rDNA loci remain compacted and are not incorporated in nucleoli. At least 10 nuclei were examined. Bar, 10 µm. **(C)** The fraction of rDNA area overlapping with nucleolin in mouse–human hybrid cell line GM15292. The area of overlap was determined between binary masks of mouse rDNA (green) and nucleolin (magenta), and between human rDNA (red) and nucleolin (magenta). Images of masks correspond to the nucleus shown in B. The graph on the right shows fractions of areas overlapping with nucleolin from 10 nuclei. A *t* test was used to compare nucleolin-overlapping area fractions of mouse and human rDNA. **, P < 0.0001.

10 chromosomal spreads examined from this hybrid cell line contained 21–34 mouse rDNA chromosomes and 1–5 human rDNA chromosomes (Fig. 6 A, left and bottom panels; and Table S1). Morphological abnormalities of mouse chromosomes such as Robertsonian fusions and mispositioned rDNA loci were present in this cell line, yet regardless of localization, 53–71% of mouse rDNA loci were active (UBF positive) and 29–47% were silent (UBF negative). All human rDNA chromosomes were UBF negative—in other words, silent (Fig. 6 A, middle panel). rDNA linkages between mouse chromosomes were generally infrequent, but in total, nine linkages were found between 175 UBF-positive, active mouse loci (one linkage per 19.4 loci on average). Importantly, all rDNA linkages occurred between mouse loci that contained UBF (Fig. 6 A, right panel), whereas zero linkages occurred between 134 mouse UBF-negative (silent) loci (Fisher’s exact P < 0.01). Human rDNA loci, all of which were UBF negative, did not have linkages in any spreads examined (zero linkages between the total of 28 human rDNA loci, not statistically significant by Fisher’s exact test).

In interphase, immuno-FISH with probes for human and mouse rDNA and antibody to nucleolar marker nucleolin revealed that on average 70% of the mouse rDNA area overlapped with nucleolin, indicating a high level of colocalization between mouse rDNA and a nucleolar marker. In contrast, human rDNA loci showed a compact morphology and only 20% overlap with nucleolin, indicating that the silent human rDNA loci tend to be excluded from the nucleolus (Fig. 6, B and C). Together with the UBF analysis, these data suggest that in this mouse–human hybrid cell line, rDNA linkages form preferentially between active mouse loci within nucleoli. Since associations between silent, UBF-negative nonnucleolar human rDNA loci were not observed, we reasoned that rDNA linkages occur primarily between active rDNA regions located inside the nucleolus.

**Figure 7.**
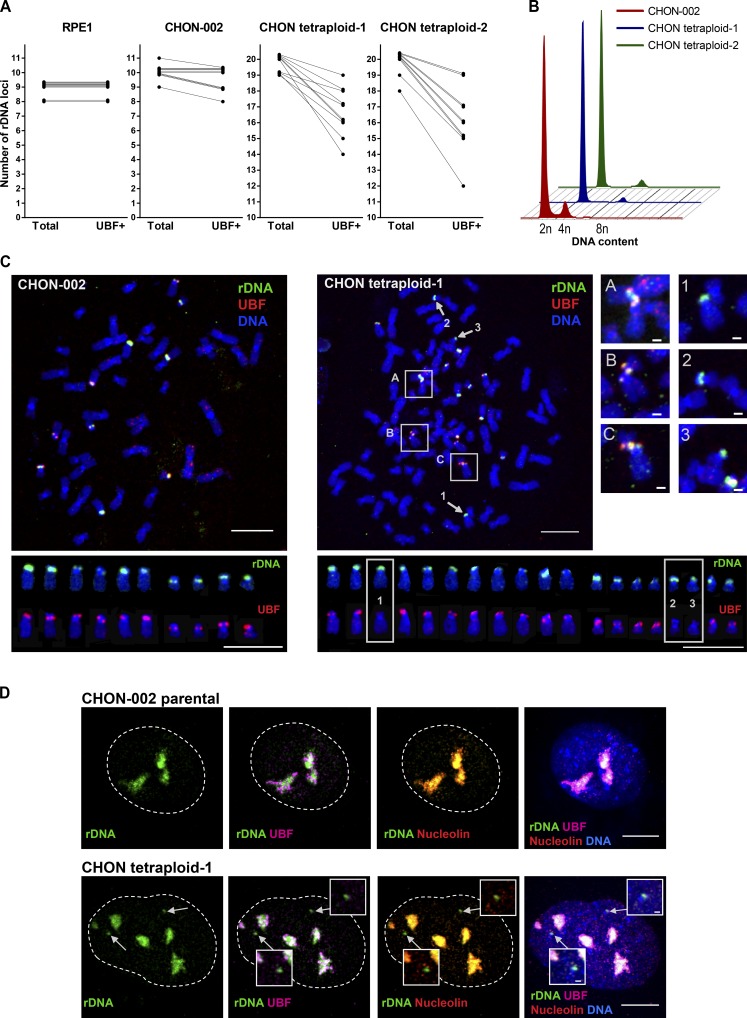
**Tetraploid derivatives of human hTERT CHON-002 cell line inactivate some rDNA loci.** These UBF-negative rDNA loci are not incorporated in nucleoli and do not form rDNA linkages. **(A)** Quantification of total number of rDNA loci and active (UBF^+^) number of rDNA loci in RPE1 and CHON cell lines. Lines between data points connect total and UBF^+^ rDNA loci in the same chromosomal spread. In RPE1, all rDNA loci were UBF positive in all cells. In most parental CHON-002 cells, all rDNA loci were UBF positive, with a few cells silencing one rDNA locus. In tetraploid CHON derivatives, all cells had some silenced rDNA loci. 10 spreads per cell line were analyzed. **(B)** Ploidy analysis of asynchronously growing tetraploid single-cell clones, designated CHON tetraploid-1 and CHON tetraploid-2, generated from hTERT CHON-002 cell line. Cells were fixed and stained with propidium iodide. FACS profiles of CHON tetraploid-1 and CHON tetraploid-2 indicate the doubling of the DNA content. **(C)** Chromosome spreads from diploid CHON-002 and tetraploid CHON tetraploid-1 cells were labeled by immuno-FISH with rDNA probe (green) and UBF antibody (red). Boxes A–C highlight rDNA linkages that were formed between UBF^+^ rDNA loci (magnified on the right; bar, 1 µm). Arrows 1–3 point to rDNA chromosomes lacking UBF. Panels below show individual rDNA chromosomes labeled with rDNA probe (top row) and UBF antibody (bottom row). In the spread from the parental CHON-002 cell line, there were 10 rDNA chromosomes, all UBF positive. In the spread from the CHON tetraploid-1 derivative, there were 19 rDNA chromosomes, 16 UBF^+^ and 3 UBF^−^ (boxes 1–3, magnified on the right). All rDNA linkages were formed between UBF-positive rDNA chromosomes. Bar, 10 µm. **(D)** Interphase nuclei from diploid CHON-002 (top) and tetraploid CHON tetraploid-1 (bottom) cells were labeled by immuno-FISH with rDNA probe (green), nucleolin antibody (red), and UBF antibody (magenta). Bar, 10 µm. In the parental CHON-002 cell nucleus, all rDNA was decompacted and associated with UBF and nucleolin. In the tetraploid derivative nucleus, compact rDNA loci were present (arrows) that were UBF negative and were not incorporated in nucleoli (magnified insets; bar, 1 µm).

To further test this idea, we used our unexpected observation that in tetraploid cell lines a small proportion of rDNA loci can be silenced. rDNA silencing is a mechanism of rRNA gene dosage control that also takes place in nonhybrids, reducing the number of active rRNA genes to match the cellular requirement for ribosome production (Grummt and Pikaard, 2003; McStay and Grummt, 2008). Previously, we derived several stably tetraploid single-cell clones from hTERT-immortalized RPE1 and CHON-002 cell lines (Potapova et al., 2016). In parental diploid RPE1 and tetraploid RPE1 cell lines, all rDNA loci contained UBF. In parental diploid CHON-002 cells, all rDNA loci were UBF positive in 5 of 10 examined chromosomal spreads, and the rest of the spreads contained one inactive rDNA locus. However, in two adapted clonal tetraploids derived from CHON-002 (designated CHON tetraploid-1 and CHON tetraploid-2), the number of UBF-positive active rDNA loci was variable but lower than the total number of rDNA loci in all chromosomal spreads examined (Fig. 7 A and Table S2). The tetraploid DNA content in these cells was stable (Fig. 7 B). An ordinary chromosomal spread from the parental diploid CHON-002 cell line contained 10 acrocentric UBF-positive rDNA chromosomes (Fig. 7 C, left). However, chromosomal spreads from tetraploid CHON derivatives consistently showed multiple rDNA chromosomes lacking UBF (Fig. 7 C, right; and Fig. S4 A). Overall, in all examined diploid and tetraploid spreads from CHON cells, 37 rDNA linkages were detected between 421 active, UBF-positive rDNA loci and zero rDNA linkages were detected between 72 inactive, UBF-negative rDNA loci (Fisher’s exact P < 0.01). Consistent with observations in the mouse–human hybrid cells, rDNA linkages in human CHON fibroblasts formed only between UBF-positive rDNA loci and were not observed among UBF-negative, silent loci.

In interphase nuclei from tetraploid CHON cells labeled by immuno-FISH with rDNA probe and antibodies to UBF and nucleolin, we observed silenced, UBF-negative rDNA loci that were compact and not incorporated in nucleoli (Fig. 7 D and Fig. S4 B). In RPE1 cells, where all rDNA loci are active, nonnucleolar rDNA loci were not observed (Fig. S4 C). We counted the number of rDNA spots that were completely nonoverlapping with nucleolar labeling to quantify rDNA loci not incorporated in nucleoli. The number of rDNA loci outside nucleoli was significantly higher in both tetraploid cell lines compared with the parental diploid CHON-002 cells (Fig. S4 D). This supports the idea that silent rDNA loci are not associated with the transcriptionally active rDNA in the same nuclear compartment. Therefore, tetraploid derivatives of a human diploid CHON-002 cell line showed that active rDNA loci can form interchromosomal linkages, but inactive rDNA loci in the same cell can be excluded from nucleoli and do not form rDNA linkages. Although correlative, together these results suggest that nucleolar colocalization of transcriptionally active rDNA from different chromosomes may promote the formation of interchromosomal rDNA linkages.

### rDNA linkages may be generated and resolved by topoisomerase II

**Figure 8.**
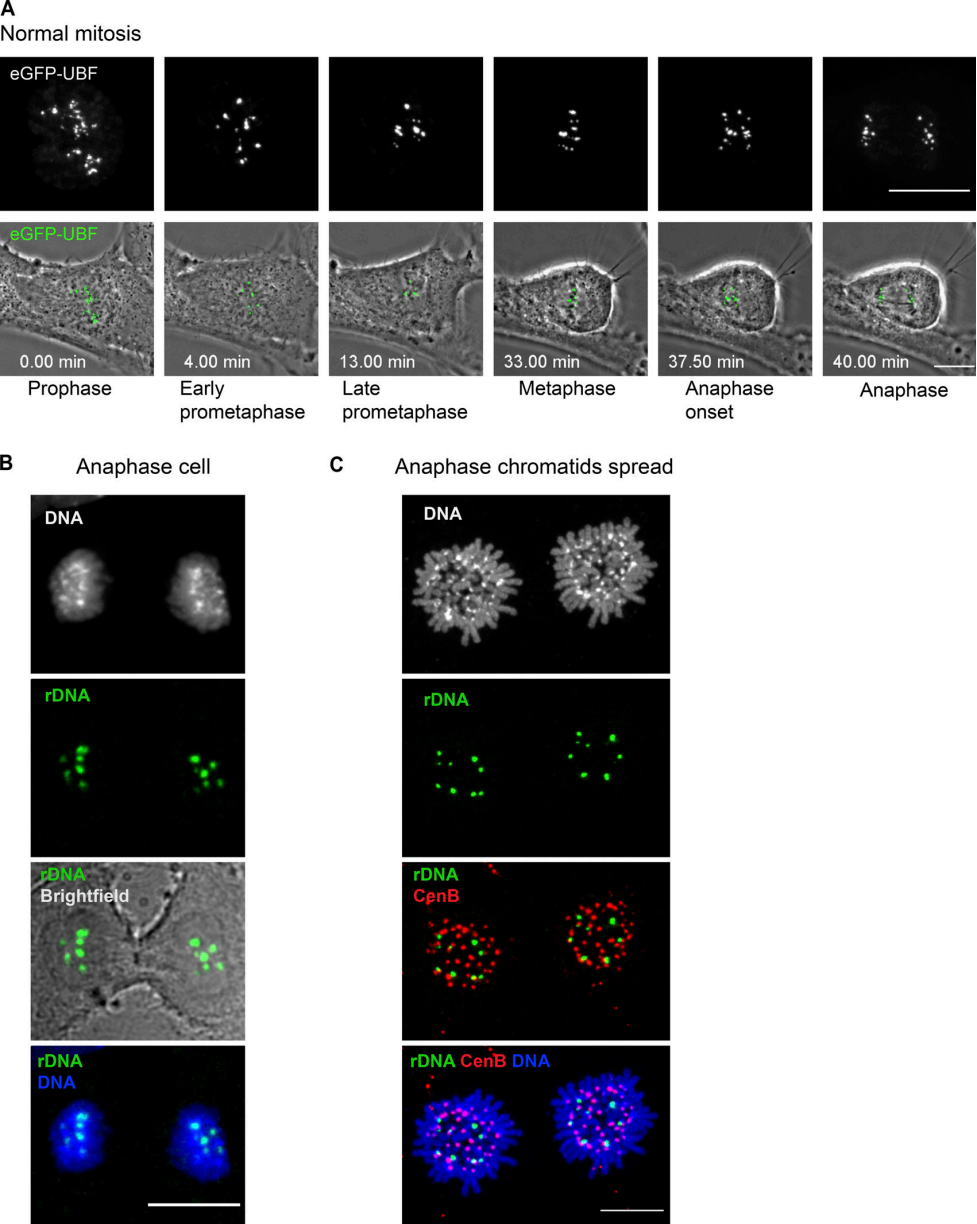
**rDNA linkages resolve at the metaphase-to-anaphase transition. (A)** Stills from time-lapse live imaging of the normal mitotic progression in an RPE1 cell expressing GFP-UBF. UBF is localized to rDNA loci that segregate in anaphase as individual dots without connections to oppositely segregating chromatids. The top panels show maximum-intensity projections of spinning disk confocal images of eGFP-UBF, and the bottom panels show maximum-intensity projections of UBF overlaid with the best focal plane phase-contrast images of the cell. The top panels are magnified 1.5× with respect to the bottom panels. Bar, 10 µm. The complete video sequence is shown in Video 1. **(B)** A normal anaphase RPE1 cell was labeled by FISH with rDNA probe (green). rDNA loci segregate as individual dots. No rDNA-containing chromatin bridges were observed. Bar, 10 µm. **(C)** A chromatid spread was prepared from an anaphase RPE1 cell and labeled by FISH with rDNA probe (green) and CenB (red). rDNA loci form distinct individual spots that do not appear to be associated with other loci. Bar, 10 µm.

What is the fate of rDNA linkages when the cell divides? All hTERT-immortalized diploid human cell lines used in this study are karyotypically stable and maintain a consistent number of rDNA chromosomes despite consistent incidences of rDNA connections in nearly every mitotic chromosomal spread. Therefore, rDNA linkages must be resolved at some point during cell division. To track the dynamics of rDNA throughout mitotic progression, we generated RPE1 cell lines stably expressing UBF fused to eGFP. UBF tagged with eGFP localizes to rDNA loci similarly to endogenous protein and is a great rDNA marker for live-cell imaging (Dundr et al., 2002; Chen et al., 2005). Time-lapse live imaging of unperturbed mitosis using eGFP-UBF–expressing cells showed that rDNA loci completely separated at the onset of anaphase (Fig. 8 A and Video 1). No lagging rDNA chromosomes or UBF-containing chromatin bridges were observed during unperturbed anaphase. All rDNA loci segregated equally to daughter cells. Consistently, rDNA FISH in normally dividing cells did not show evidence of lagging rDNA loci or rDNA chromatin bridges between the daughter cells (Fig. 8 B).

To determine if interchromosomal rDNA linkages persisted between separated chromatids in anaphase, we prepared chromosomal spreads from anaphase cells and labeled rDNA by FISH (Fig. 8 C). Enrichment for anaphase cells was done by presynchronizing cells in the G2 phase of the cell cycle with the Cdk1 inhibitor RO-3306 (Vassilev et al., 2006), followed by release into the medium without the drug. After release, when cells were in various stages of mitosis, they were collected and swollen in hypotonic buffer followed by fixation. In this case, individual chromatids of anaphase cells were separated well enough to distinguish individual rDNA loci. rDNA formed distinct individual spots that did not show associations with one another. Together, these data imply that most inter- and intrachromosomal rDNA linkages present among metaphase chromosomes resolve at the metaphase-to-anaphase transition.

**Figure 9.**
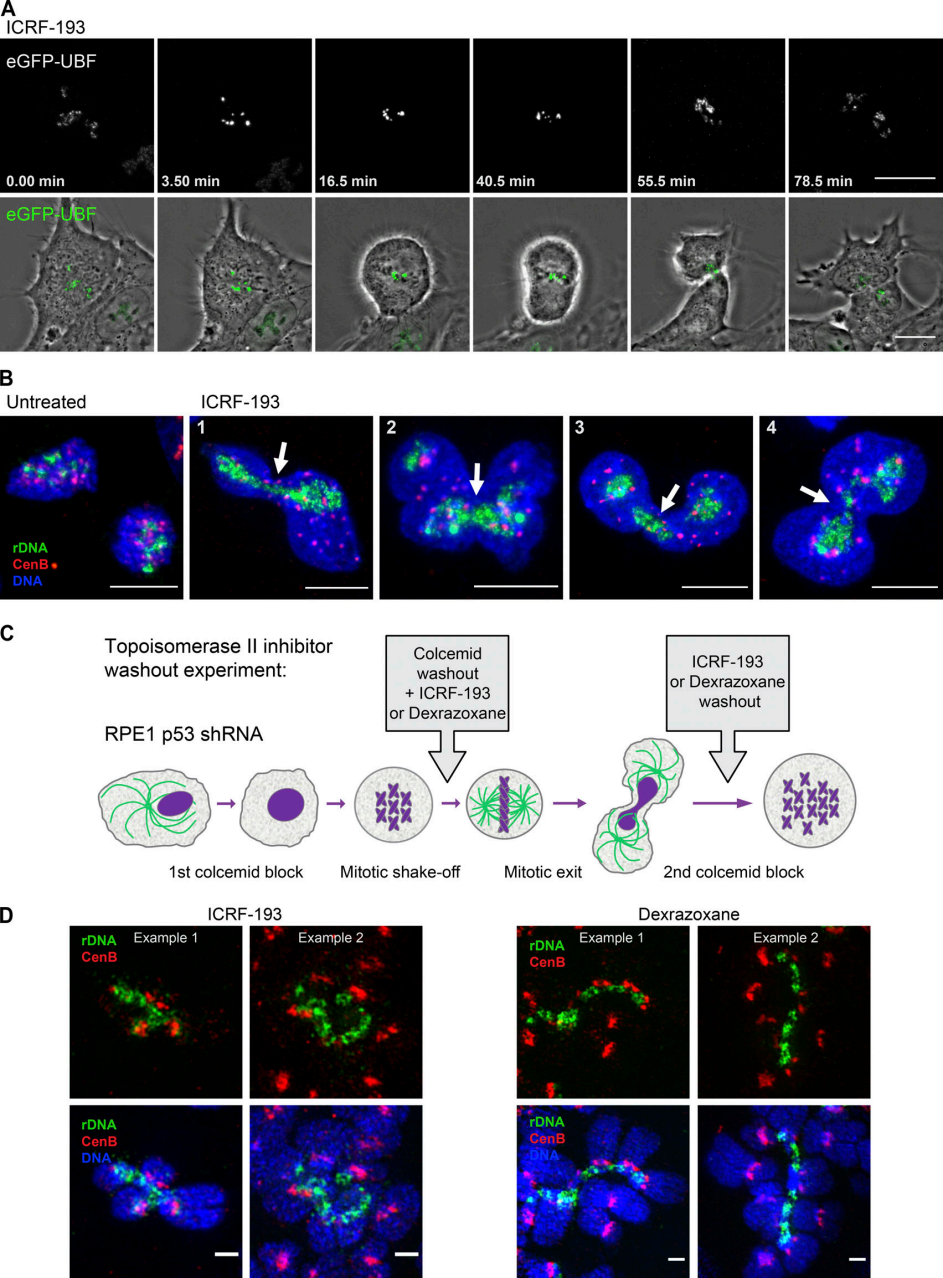
**Inhibition of topoisomerase II in mitosis prevents the resolution of rDNA linkages at the metaphase-to-anaphase transition. (A)** Time-lapse live imaging of the mitotic progression in RPE1 cells expressing GFP-UBF treated with 5 µM topoisomerase inhibitor ICRF-193. The cell treated with topoisomerase inhibitor shows impaired sister chromatid segregation and fails to segregate rDNA marked by GFP-UBF. The drug was added shortly before the initiation of imaging. The top panel shows maximum-intensity projections of spinning disk confocal images of GFP-UBF, and the bottom panel shows maximum-intensity projections of UBF overlaid with the best focal plane phase-contrast images of the cell. The top panel is magnified 1.5× with respect to the bottom panel. Bar, 10 µm. The complete video sequence is shown in Video 2. **(B)** Localization of rDNA and centromeres in cells that divided in the presence of topoisomerase inhibitor ICRF-193. Asynchronously growing c-Myc–overexpressing RPE1 derivative cell line cMyc 3 was untreated (first left panel) or treated with 5 µM ICRF-193 for 30 min (right panels). Cells were fixed and labeled by FISH with rDNA probe (green) and CenB probe (red). DNA was counterstained with DAPI. Maximum-intensity projections of confocal images depict cells that failed to segregate the rDNA. Four examples of individual cells are shown (1–4). Arrows point to rDNA trapped in the cleavage furrow. Bar, 10 µm. **(C)** A schematic of the experimental design of topoisomerase II washout experiment is shown. RPE1 cells stably expressing p53 shRNA were arrested in mitosis by colcemid for 10 h and collected by mitotic shake-off. Colcemid was removed, and 10 µM ICRF-193, 500 µM dexrazoxane, or control vehicle (DMSO) were added to the mitotic cells. Cells were allowed to rebuild the mitotic spindle, exit mitosis, and attach to the plates for 4 h. After this, topoisomerase inhibitors were washed out, and cells were allowed to progress through the cell cycle for another 14–15 h. Therefore, cells were treated with topoisomerase II inhibitors during mitotic exit only. Then, colcemid was added again for 10 h to collect cells in the next mitosis for chromosomal spreads. **(D)** Cells that exited mitosis in the presence of topoisomerase II inhibitors display complex rDNA linkages. Mitotic spreads prepared from cells in C were labeled by FISH with rDNA probe (green) and CenB probe (red) and imaged by SIM. Fragments of spreads containing complex multichromosomal linkages are shown. Two examples are shown per drug treatment. Bar, 1 µm.

What could be the mechanism of resolution of rDNA linkages in anaphase? Two mechanisms are responsible for disengagement of sister chromatids at the metaphase-to-anaphase transition: cleavage of the cohesin subunit Rad21 by separase (Uhlmann et al., 1999; Haarhuis et al., 2014) and resolution of sister chromatid intertwines by type II topoisomerases (Gorbsky, 1994; Nitiss, 2009). Knocking down *RAD21* by siRNA did not affect rDNA linkages: they persisted even though sister chromatids separated in mitotically arrested cells (Fig. S5 A). This showed that rDNA linkages are maintained in the absence of cohesion and that cohesion resolution is not sufficient to resolve them. On the other hand, chemical inhibition of type II topoisomerases α and β with catalytic inhibitors ICRF-193 (Fig. 9 A and Video 2) or dexrazoxane (Fig. S5 B and Video 3) in dividing RPE1 cells stably expressing eGFP-UBF prevented segregation of rDNA loci. Although chromosome segregation was generally compromised by topoisomerase inhibitors, rDNA tended to be trapped in the cleavage furrow (Fig. 9 B and Fig. S5 C), suggesting that segregation of rDNA relies heavily on the function of topoisomerases.

Inhibition of topoisomerases does not prevent activation of separase and cleavage of cohesin but is known to interfere with sister chromatid segregation (Gorbsky, 1994; Ishida et al., 1994). Topoisomerase inhibitors applied to interphase cells also cause interphase cell cycle arrest, and this precluded preparation of mitotic spreads from cells pretreated with these drugs. To examine consequences of failure to resolve rDNA linkages in anaphase, we designed an experimental strategy in which the resolution of rDNA loci was prevented by inhibiting topoisomerases only during mitotic exit. Cells were arrested in mitosis with the mitotic spindle poison colcemid, which was then washed out, and topoisomerase inhibitors ICRF-193 or dexrazoxane were added to the cells. This allowed cells to rebuild a mitotic spindle and exit mitosis without the decatenating activity of type II topoisomerases. After cells exited mitosis, all inhibitors were washed out to allow progression through the next cell cycle. Then these cells were collected in the subsequent mitosis and analyzed by rDNA FISH (Fig. 9 C).

For this experiment, we used RPE1 cells stably expressing a shRNA against p53 (Potapova et al., 2016), because cells with functional p53 arrest in the G1 stage of the cell cycle after prolonged mitotic arrest (Uetake and Sluder, 2010). The karyotype of these p53-knockdown RPE1 cells is aneuploid with varying numbers of rDNA chromosomes, but the incidence of spontaneous rDNA linkages is not high. Most of the cells that exited mitosis in the presence of topoisomerase II inhibitors became tetraploid. Since these cells were aneuploid to start with, the total number of rDNA linkages in chromosomal spreads was not very informative. Yet importantly, rDNA displayed strikingly complex linkages, connecting together four or more chromosomes (Fig. 9 D). Notably, there were no linkages observed between centromeres located on the same chromosomes, suggesting that rDNA resolution is more reliant on topoisomerase activity than centromeres. At least 10 spreads were examined from each of the three conditions: ICRF-193, dexrazoxane, or vehicle control. Complex or “super” linkages were observed only in drug-treated cells. We suggest that the super rDNA linkages represent persistent unresolved topological linkages plus new linkages that form during the subsequent cell cycle.

**Figure 10.**
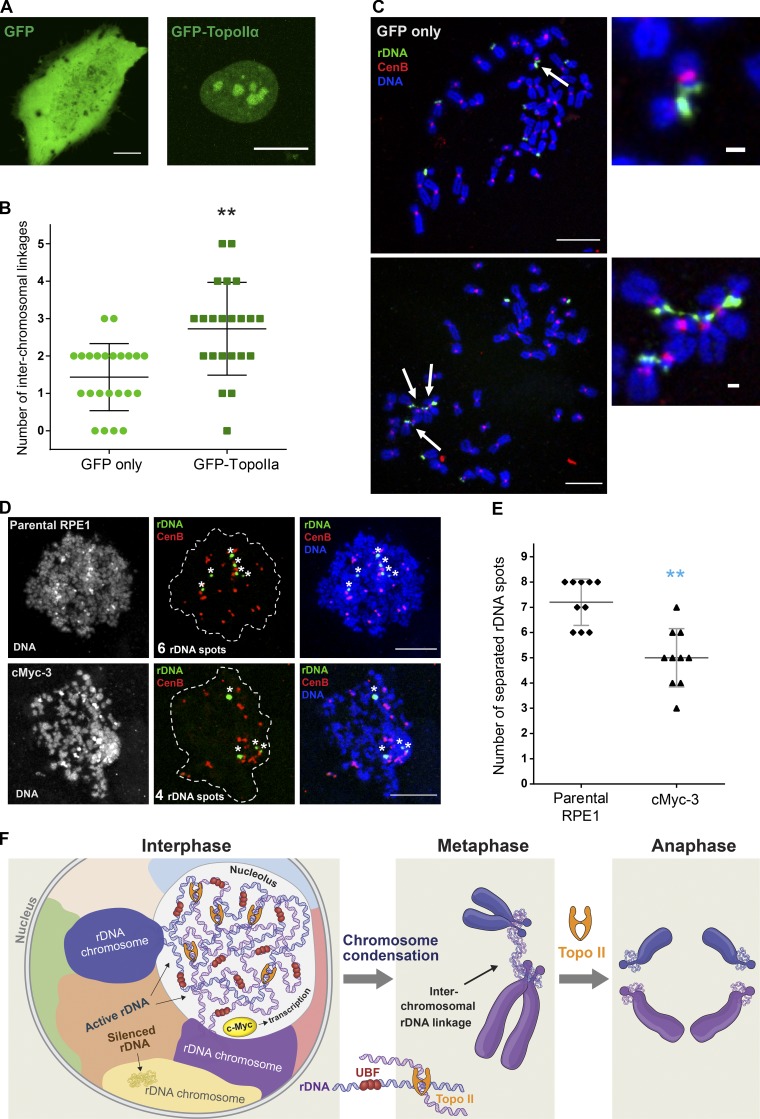
**Topoisomerase IIα plays a major role in creating and resolving rDNA linkages. (A)** Fluorescent images of interphase cells transiently transfected for 24 h with GFP-topoisomerase IIα (TopoIIα; top) or GFP only (bottom) are shown. GFP-TopoIIα shows propensity to localize in nucleoli. Bar, 10 µm. **(B)** The number of interchromosomal rDNA linkages in chromosomal spreads from cells transiently transfected with GFP or GFP-TopoIIα was quantified. For this experiment, RPE1 cells were transfected with indicated plasmids for 24 h and arrested in mitosis by addition of colcemid for 12–14 h. Mitotic cells were collected by shake-off and FACS sorted to isolate GFP-positive mitotic cells. Mitotic spreads from cells expressing GFP-TopoIIα show significantly more rDNA linkages compared with GFP only. The Mann–Whitney *U* test was used to compare GFP-TopoIIα–expressing samples with GFP-expressing control. **, P = 0.0002. **(C)** Representative confocal images of chromosome spreads from cells transiently transfected for with GFP-TopoIIα, GFP only (top), or TopoIIα (bottom). Spreads were labeled by FISH with rDNA probe (green) and CenB probe (red). Bar, 10 µm. Arrows point to rDNA linkages shown in magnified insets on the right; bar, 1 µm. **(D)** Chromatin spreads from interphase parental RPE1 cells (top) and cMyc-3 derivative (bottom) cells. Interphase cultures were treated with 100 nM calyculin A for 90 min to induce premature chromatin condensation and labeled by FISH with rDNA probe (green) and CenB probe (red). Asterisks denote individual separated rDNA spots. Bar, 10 µm. **(E)** Quantification of the number of separated rDNA spots in 100 nM calyculin A–treated interphase cells (D) labeled by FISH with rDNA probe. High-resolution confocal images of ≥10 condensed chromatin spreads were examined. The Mann–Whitney *U* test was used to compare c-Myc–overexpressing samples with parental RPE1. **, P < 0.001. **(F)** Working model for how interchromosomal rDNA linkages are generated and resolved. Inter- and intrachromosomal rDNA catenations may form due to the intertwining of transcriptionally active DNA from different chromosomes in the crowded nucleolar environment. Formation of rDNA linkages depends on UBF, and boosting rDNA transcription by overexpressing c-Myc increases the frequency of rDNA linkages. Silent (UBF^−^) loci are not incorporated in nucleoli and do not form linkages. Topoisomerase II generates more catenations by trying to correct the local topology in the interphase nucleus but then resolves these catenations during the metaphase–anaphase transition. Therefore, transcription-dependent formation of rDNA linkages does not lead to chromosomal missegregation and genomic instability under normal conditions.

Transient overexpression of topoisomerase IIα tagged with GFP in RPE1 cells also led to an increase in rDNA linkages. Ectopically expressed GFP-topoisomerase IIα localized to interphase nuclei with a strong propensity for nucleoli (Fig. 10 A). Mitotic chromosomal spreads collected from cells overexpressing GFP-topoisomerase IIα for 24 h showed more interchromosomal rDNA linkages than cells overexpressing GFP alone (Fig. 10, B and C). This suggests that topoisomerase IIα may create rDNA intertwines in interphase, which manifest as rDNA linkages in mitosis, before being resolved in anaphase by the same enzyme. Thus, the topological activity of topoisomerase II is potentially responsible for both rDNA linkage formation in interphase and resolution during mitosis.

To examine whether rDNA linkages exist in interphase, we induced premature DNA condensation and mitosis-like state in interphase RPE1 cells with the phosphatase inhibitor calyculin A (Stevens et al., 2010). Calyculin A treatment of interphase cells induced chromatin condensation and nuclear envelope breakdown, permitting preparation of chromatin spreads from these cells and analysis of the number of condensed rDNA loci (Fig. 10 D). If rDNA loci were not linked in interphase, the expected number of separated rDNA spots would be 9, since RPE1 cells have rDNA loci on 9 of 10 acrocentric chromosomes. However, the observed number of individual rDNA spots was fewer than nine. In parental RPE1 cells, six to eight rDNA spots were generally observed, while only three to seven spots were observed in the c-Myc–overexpressing line (Fig. 10 E). The observation of fewer than nine separated spots indicates that some spots contain rDNA loci from more than one chromosome. In other words, these loci were linked in interphase before calyculin A–induced DNA condensation. The lower number of separated spots in c-Myc–overexpressing cells is consistent with the higher frequency of rDNA linkages observed in chromosome spreads.

## Discussion

The existence of interchromosomal associations between short arms of acrocentric chromosomes was first noted by pioneering cytogenetics studies in the 1960s (Ferguson-Smith and Handmaker, 1961), but their basis has never been explained. Superresolution imaging allowed us to unambiguously detect physical inter- and intrachromosomal DNA connections. Furthermore, we speculate that associations may represent catenation events that occur as a consequence of transcriptional activity and colocation of rDNA regions from heterologous chromosomes in the same nucleolus.

Interestingly, physical “linkers” in the form of thin, highly extensible filaments between chromosomes have been observed in classic microdissection experiments, where chromosomes were mechanically pulled out of live mitotic cells. It remains controversial whether these filaments consist of chromatin and/or other structures, although they are sensitive to digestion by nucleases (Maniotis et al., 1997; Poirier and Marko, 2003; Marko, 2008). The DNA sequences involved in these interchromosomal linkers are unknown, but our results suggest that rDNA could be a linker sequence.

We provide evidence that both the formation and resolution of rDNA linkages depend on topoisomerase II. If linkages constrain chromosome organization, they may represent a method for connecting the transcription of the rDNA, which is modulated by nutritional signals and major proliferative and stress pathways, to the large-scale organization of the genome. Given the ubiquitous reports of chromosomal associations in the literature, these linkages may be a pervasive governing feature of chromosome organization in many cell types. Transcription generates torsional stress that can entangle DNA by causing supercoiling ahead of and behind the transcription machinery. Type II topoisomerases manage DNA supercoiling during transcription by breaking and rejoining DNA strands (Pommier et al., 2016). Since the relief of DNA supercoiling is important for supporting high levels of transcriptional activity at the rDNA, we speculate that topoisomerase activity may also entangle neighboring DNA strands, especially given the intermingling of rDNA from different chromosomes within the same nucleolus.

Our proposed model is that rDNA linkages are interchromosomal catenations that may be a consequence of intense transcription in a crowded nucleolar environment. DNA catenation of sister chromatids plays an important role in holding them together (Bauer et al., 2012; Sen et al., 2016), and we speculate that rDNA linkages in interphase could be holding the nucleolus together. Type II topoisomerases may create rDNA linkages while in the process of relieving local torsional stress from transcription but then resolve linkages during the metaphase-to-anaphase transition (Fig. 10 F). For this reason, interchromosomal rDNA linkages do not lead to chromosomal missegregation and do not normally cause genomic instability. In other words, these linkages are not pathological per se. However, short arms of the acrocentric chromosomes are the sites of chromosomal fusions in the formation of Robertsonian translocations, a frequent structural chromosomal rearrangement in humans (McKinlay Gardner et al., 2011). We speculate that rDNA linkages and formation of Robertsonian translocations are related. First, unresolved linkages may cause a chromosome break, potentiating translocation. Second, the physical proximity afforded by the linkages may predispose acrocentric chromosomes to translocation by unrelated break events. Our findings suggest that transcriptionally active DNA loci form physical interchromosomal connections that can shape the genome organization.

## Materials and methods

### Cell culture

Isogenic HMECs 184DTERT, 184B5ME, 184AA3, 184p16sMY, and 184FMY2 (Stampfer and Bartley, 1985; Stampfer et al., 2001, 2003; Garbe et al., 2014) were cultured for chromosomal spread preparation in M87A medium (Garbe et al., 2009) supplemented with 0.1% AlbuMAX I (Invitrogen), 0.5 ng/ml cholera toxin (Sigma-Aldrich), and 0.1 nM oxytocin (Sigma-Aldrich). hTERT RPE1 and hTERT CHON-002 (ATCC) were grown at 37°C in 5% CO_2_ in DMEM-F12 medium supplemented with 10% FBS. HFF-1s (ATCC) were grown in DMEM supplemented with 15% FBS. Human iPSCs DYS0100 (ATCC) were cultured on CellMatrix Gel–coated dishes without a feeder layer in Pluripotent Stem Cell SFM XF/FF medium as recommended by ATCC. LoVo cells (ATCC) were grown in F-12K medium supplemented with 10% FBS. NCI-H209 cells (ATCC) were cultured in RPMI 1640 supplemented with 10% FBS. Human–mouse somatic cell hybrid cell line GM15292 was obtained from the Coriell Institute and cultured in high-glucose DMEM with 2 mM GlutaMax (Thermo Fisher Scientific) and 10% FBS. Mouse V6.5 embryonic stem cells (Novus Biologicals) were grown in feeder-free conditions using N2B27+2i medium supplemented with 2,000 U/ml of leukemia inhibitory factor (Millipore), 3 µM CHIR99021 (Tocris), and 1 µM PD0325901 (SCT). Cells were grown on gelatinized tissue culture plates without a feeder layer. Chromosomal spreads from human male lymphocytes were obtained from Applied Genetics Laboratories. Proliferating tetraploid CHON-002 cells were generated from diploid hTERT CHON-002 cells (ATCC). Briefly, asynchronously growing cells were synchronized by double thymidine (2 mM) block followed by three cycles of 12-h incubation with 0.1 µg/ml of nocodazole. Mitotic shake-off was performed at the last nocodazole treatment to enrich for mitotic cells. 22 h after the last nocodazole treatment, single cells were sorted into 96-well plates and allowed to grow for 3–4 wk. DNA content analysis by FACS was performed to assess ploidy. Tetraploidy was confirmed by analyzing chromosome spreads.

### Chromosome spreads, FISH, and immuno-FISH

For chromosome spread preparation, cells were arrested in mitosis by the addition of KaryoMAX Colcemid solution (0.1 µg/ml; Life Technologies) for 4–8 h followed by harvesting using trypsin. Human HFF-1 cells and iPSCs were arrested in mitosis by incubation in 100 µM monastrol (Tocris Bioscience) and 10 µM ProTAME (BostonBiochem) because prolonged colcemid incubation was toxic to these cells. Trypsinized cells were collected by centrifugation for 5 min at 300 *g* and gently resuspended in a small amount of medium (∼1 ml). Resuspended cells were swelled for 7–10 min in 0.4% KCl solution at room temperature and prefixed by the addition of freshly prepared methanol:acetic acid (3:1) fixative solution (∼100 µl per 10 ml total volume). Prefixed cells were collected by centrifugation and fixed in methanol:acetic acid (3:1) fixative solution. Spreads were dropped on a glass slide or #1.5 glass coverslip and incubated at 65°C for ≥2 h. For anaphase spread preparation, cells were presynchronized with 10 µM RO-3306 (Tocris) overnight and released into the medium without the drug. 40–60 min after release, cells were collected by trypsinization and processed as above. Before hybridization, slides were treated with 1 mg/ml RNase A (1:100; Qiagen) in 2× SSC for ≥45 min at 37°C and dehydrated in a 70, 80, and 100% ethanol series for 2 min each. Slides were then denatured in 70% formamide/2× SSC solution preheated to 72°C for 1.5 min and immediately cooled to −20°C. Labeled DNA probes were denatured separately in hybridization buffer (Empire Genomics) by heating to 80°C for 10 min before applying to denatured slides.

For FISH in asynchronously growing cultures, cells were grown in chambered glass slides (Thermo Fisher Scientific) and fixed in 2% PFA in PBS (30 min). They were permeabilized with 0.5% saponin and 0.5% Triton X-100 for 30 min and treated with RNase as above. Then cells were pretreated with 0.1 N HCl for 15 min, washed in 2× SSC buffer, and preincubated in 50% formamide/2× SSC for ≥30 min before denaturation and hybridization. In this case, specimens and probes were denatured together for 7 min at 85°C.

Fluorescein-labeled probes for human rDNA (BAC clone RP11-450E20) and mouse rDNA (BAC clone RP23-225M6) were obtained from Empire Genomics. Cy-3–labeled probes for telomeres (TelC) and centromeres (CenB) were obtained from PNA Bio. Specimens were hybridized to the probe under a glass coverslip or HybriSlip hybridization cover (Grace Biolabs) sealed with rubber cement or Cytobond (SciGene) in a humidified chamber at 37°C for 48–72 h. After hybridization, slides were washed in 50% formamide/2× SSC three times for 5 min per wash at 45°C, then in 1× SSC solution at 45°C for 5 min twice and at room temperature once. Slides were then rinsed with double-deionized H_2_O, air dried, and mounted in Vectashield containing DAPI (Vector Laboratories).

For immuno-FISH, chromosome spreads fixed in methanol:acetic acid were used, and slides labeled by FISH were first subjected to antigen unmasking in hot (65°C) citrate buffer, pH 6.0, for 1 h before processing for immunofluorescence. PFA-fixed slides did not require antigen unmasking. Slides were blocked with 5% boiled normal goat serum or BSA in PBS/0.5% Triton X-100. Primary and secondary antibodies were diluted in 2.5% (wt/vol) BSA/PBS/0.5% Triton X-100. Specimens were incubated with primary antibody at a minimum overnight, washed three times for 5 min, incubated with secondary antibody also overnight, and washed again three times for 5 min. All washes were performed with PBS/0.5% Triton X-100. Vectashield containing DAPI was used for mounting. Confocal images were acquired using a LSM710 confocal microscope (Zeiss) with a 63×/1.40-NA oil objective. At least 10 cells were imaged for each condition. Data were visualized and analyzed using Fiji (National Institutes of Health). To quantify the fraction of rDNA overlapping with nucleolin in the mouse–human hybrid cell line, nucleolar segmentation was performed by Otsu thresholding of maximum-intensity projections of the nucleolin channel after background subtraction with a radius of 50 pixels and blurring with an SD of 4 pixels. Objects <1 µm^2^ were eliminated. Maximum-intensity projections of mouse and human rDNA channels were also segmented by thresholding, and binary masks of each channel were generated. The area of overlap between mouse rDNA and nucleolin masks, and between human rDNA and nucleolin masks, was determined using the AND operation in ImageJ.

### SIM and quality control

SIM images were acquired on an Applied Precision OMX Blaze microscope (GE Healthcare) equipped with three PCO Edge sCMOS cameras, each dedicated to one specific channel. An Olympus 60× 1.42-NA Plan Apo N oil objective was used. SIM reconstruction was performed with the Applied Precision SoftWoRx software package (GE Healthcare) following the Applied Precision protocols. Color alignment of XY direction was performed using the color alignment slide provided by GE Healthcare. SIM images were reconstructed using SoftWoRx software (GE Healthcare) with a Wiener filter of 0.001. Immersion oils with a refractive index of 1.512–1.516 were chosen to optimize SIM image quality. The refractive index value was calculated and determined using the lens information tool in SoftWoRx 6.5.2 (GE Healthcare). To obtain corresponding wide-field images, the light path was set to conventional illumination settings. The SIMcheck analysis was performed as described in Ball et al. (2015).

Pearson colocalization measurements were performed similarly to the analysis described in Slaughter et al. (2013). Briefly, line profiles were drawn repeatedly through the rDNA regions and averaged over a 3-pixel width to reduce noise but not average out heterogeneity. The resulting profiles were subjected to Pearson correlation analysis and then repeatedly shifted relative to one another to create a spatial correlation profile. Multiple rDNA loci were averaged together to obtain average Pearson profiles. Custom ImageJ plugins for this analysis can be downloaded from http://research.stowers.org/imagejplugins/.

### STED microscopy

STED images were acquired with a 100×, 1.4-NA oil objective on a Leica SP8 Gated STED microscope. Carboxytetramethyl rhodamine–labeled rDNA was excited with a pulsed white light (80 MHz) tuned to 594 nm and depleted with 660-nm continuous laser at 80–90% maximum power. All images were acquired in 2D mode to maximize lateral resolution, and each image was averaged eight times in line average mode. The emission photons were collected with internal Leica HyD hybrid detector with a time gate of 0.6–6 ns. Raw STED images were deconvolved with Huygens professional deconvolute software (version 14.10; Scientific Volume Imaging). A theoretical estimated point spread function was calculated from the raw STED image. During deconvolution, all parameters were set by default, but the background was set to the value measured from raw data, and the signal-to-noise was set in the range of 12–15 depending on the intensity of the signal.

### Live-cell imaging

For live-cell imaging, cells were grown on 35-mm ibiTreat µ-dishes (Ibidi). Time-lapse Z-stack images were captured on a Nikon TiE microscope equipped with a 60× phase-contrast objective, 1.4-NA, Perfect Focus mechanism, Yokogawa CSU-W1 spinning disk, and Flash 4.0 sCMOS camera. Cells were imaged in the regular growth medium; 37°C and 5% CO_2_ was maintained using an environmental control chamber (Okolab). 10 µM ICRF-193 (Santa Cruz Biotechnology) and 500 µM dexrazoxane (TCI America) were added shortly before initiation of imaging. Images were acquired with NIS Elements software. Image processing (maximum-intensity projection, background subtraction, and stack combining) was done in Fiji.

### 5-EU labeling

For 5-EU incorporation assays, cells were typically seeded in multiwell black optically clear-bottom tissue culture–treated plates (Perkin Elmer) and treated with 1 mM 5-EU (Thermo Fisher Scientific) for 3 h. Cells were fixed in 4% PFA/PBS for 15 min and permeabilized with 0.5% Triton X-100. Fixed cells were washed with PBS and stained with 1 µM Alexa Fluor 555–conjugated azide diluted in PBS containing 2 mM CuSO_4_ and 50 mM ascorbic acid. To counterstain the DNA, Hoechst 33342 (Sigma-Aldrich) was added to 2 µg/ml. Cells were incubated for several hours or overnight at room temperature protected from the light and evaporation, then washed three times with PBS. 5-EU–labeled RNA fluorescence was quantified by high-throughput imaging using Celigo Imaging Cytometer (Nexcelom Bioscience). Four fields of view containing thousands of cells were analyzed to determine mean parameters and SD.

### Plasmid and siRNA transfections

A plasmid encoding the wild-type human UBF gene tagged with eGFP (pEGFP-C1-UBF) was obtained from Addgene (Chen and Huang, 2001; plasmid 26672; Addgene). The plasmid encoding GFP only was the pAcGFP1-N1 vector from Clontech. The plasmid encoding wild-type human topoisomerase IIα tagged with GFP was supplied courtesy of G.J. Gorbsky, Oklahoma Medical Research Foundation, Oklahoma City, OK (Daum et al., 2009). For generating a stable cell line overexpressing eGFP-UBF, RPE1 cells were transfected using X-tremeGENE 9 DNA Transfection Reagent (Roche) according to the manufacturer’s directions. Transfected cells were selected with 1 mg/ml G418 (A.G. Scientific). For generating RPE1 cells stably overexpressing human c-Myc, cells were seeded at low confluence and transduced with premade lentiviral particles containing the c-Myc gene (product FCT019; Kerafast) in the presence of 10 µg/ml polybrene (EMD Millipore). Single-cell colonies were selected with 10 µg/ml puromycin (InvivoGen) and isolated using Scienceware polystyrene cloning cylinders (Sigma-Aldrich). For generating RPE1 cells stably expressing p53 shRNA, cells were transduced with premade lentiviral particles (V3LHS_333920; GE Healthcare) in the presence of 10 µg/ml polybrene.

For siRNA-mediated knockdowns, RPE1 cells were transfected with siRNA using Lipofectamine RNAiMAX (Thermo Fisher Scientific) according to the manufacturer’s directions. siRNAs targeting the indicated genes were obtained from GE Healthcare. siRNA sequences used in this article are listed in Table S3.

### DNA sequencing (DNA-seq) library preparation, sequencing, and analysis

DNA isolation was performed using the Maxwell 16 Cell DNA kit on a Maxwell 16 Research Instrument (Promega). DNA-seq libraries were generated from 100 ng of genomic DNA, which was sonicated to 350 bp using the Covaris 220 instrument. Libraries were prepared using the Sciclone G3 NGS Workstation from Perkin Elmer, the KAPA HTP Library Preparation Kit (KK8234), and the NextFLEX Bioo Scientific DNA Barcodes kit (NOVA-514104) and were purified using the Agencourt AMPure XP system (A63882; Beckman Coulter). Quality control was completed using the Agilent 2100 Bioanalyzer and the Invitrogen Qubit 2.0 Fluorometer. Libraries were requantified, normalized, pooled, and sequenced on an Illumina HiSeq 2500 instrument as a RapidSeq 100-bp paired read run using HiSeq Control Software (v2.2.58). After sequencing, Illumina Real Time Analysis version 1.18.64 was run to demultiplex reads and generate FASTQ files.

The human consensus 45S rDNA sequences were obtained from the National Center for Biotechnology Information (NCBI accession no. U13369). Raw FASTQ whole-genome DNA-seq reads were mapped to the 45S locus and a set of 16,022 preselected putative single-copy exons (the largest from each gene) using Bowtie2 v2.1.0 with default parameters. Only concordant read pairs were kept in the downstream analysis. The rDNA copy number of r45S was calculated as the mean coverage for the locus. To make samples comparable to each other, the rDNA copy number was further normalized to the background genome coverage, which is calculated as the median coverage of the single-copy exons (Gibbons et al., 2015; Xu et al., 2017a). The chromosomal coverage was calculated as the median coverage of each chromosome and was further normalized to the median of all chromosomal coverage within a sample. Each cell line was sequenced once.

### Real-time qPCR

RNA isolation was performed using Maxwell 16 LEV simplyRNA Tissue kit on a Maxwell 16 Research Instrument (Promega). Reverse-transcription reactions were performed using 2,000 ng of each RNA template using SuperScript VILO Master Mix (Thermo Fisher Scientific). Reactions containing cDNA were diluted in PerfeCTa SYBR Green FastMix with Low ROX (Quanta Biosciences) and cycled on the QuantStudio 7 Flex Real-Time PCR System (Applied Biosystems). cDNA samples from three biological replicates were measured for each cell line, and each sample was measured in three technical replicates. GAPDH was used as a normalization control. Expression of pre-rRNA was determined by an average fold change of three primer sets to the 5′ETS. Each primer pair was confirmed for linearity of amplification by analyzing standard curves. Ct values were analyzed using the 2^−ΔΔCT^ method (Livak and Schmittgen, 2001). Ct values of parental control cell lines were averaged, and the results are presented relative to the control average Ct values. Error bars denote SD. Primers used in this study are listed in Table S3.

### Flow cytometry for DNA content measurements

For DNA content analysis, cells were trypsinized, fixed in 70% ethanol, and stained with FxCycle PI/RNase Staining Solution (Thermo Fisher Scientific) according to the manufacturer’s directions. Data from labeled cells were collected on the MACSQuant flow cytometer (Miltenyi Biotec) and analyzed using FlowJo software.

### Immunofluorescence and high-throughput nucleolar measurements

For nucleolin and Ki67 immunofluorescence, cells were fixed in 4% PFA/PBS for 15 min and permeabilized with 0.5% Triton X-100. Blocking was done with 5% boiled normal goat serum or 5% BSA in PBS/0.5% Triton X-100. Primary and secondary antibodies were diluted in 2.5% BSA/PBS/0.5% Triton X-100. Specimens were incubated with primary antibodies overnight, washed three times for 5–10 min, and incubated with fluorescence-conjugated secondary antibodies for 2–4 h. All washes were performed with PBS/0.5% Triton X-100. DNA was counterstained with DAPI or Hoechst 33342 (Thermo Fisher Scientific).

Z-stack tiled images were acquired on the Nikon TiE microscope with a Yokogawa CSU W1 spinning disk and Hamamatsu Flash 4.0 camera. Maximum-intensity projections of Z-stacks were generated. The nuclear (DAPI) channel was segmented to find individual nuclei. The Ki67 signal was quantified by measuring average fluorescent intensity per nucleus. Three tiled images containing multiple cells were analyzed per cell line. Nucleolin was used to perform nucleolar area and count measurements. These measurements were performed using a multistep strategy with custom Fiji plugins available at http://research.stowers.org/imagejplugins as follows. First, the DAPI images were Gaussian blurred with a SD of 12 pixels. Next, a rolling ball was subtracted with a radius of 200 pixels. To normalize the intensities of neighboring nuclei, the DAPI image was divided by a 200-pixel SD Gaussian-blurred version of itself excluding blurred pixels less than one. Finally, DAPI objects were thresholded at an intensity of 15% of the maximum processed nuclear intensity for each image. Nuclei on the image border and nuclei with areas <5,000 pixels and >18,000 pixels were eliminated. Next, holes inside nuclei were filled to obtain the final nuclear masks. Circularity is defined as 4 × π × area/(perimeter2), which has a value of 1 for a perfect circle and 0 for any structure with zero area. To avoid partially segmented nuclei, objects with a circularity <0.8 were eliminated from further analysis. Nucleoli were segmented using a slightly different strategy. First, the nucleolar channel was Gaussian blurred with a SD of 2 pixels, after which a rolling ball background with a radius of 15 pixels was subtracted. Next, the nucleolar image was divided by a Gaussian-blurred version of itself with a blur SD of 30 pixels. Next, the nucleoli in each nucleus were thresholded at an intensity of 20% of the maximum processed intensity in each nucleus. Finally, nucleoli <4 pixels in area were eliminated. Once nucleoli were segmented, a table was generated with entries for each nucleolus, listing nuclear ID and nuclear and nucleolar statistics. Averages were calculated over nuclei and then over the entire image with SEM calculated over the entire image. Three tiled images containing multiple cells were analyzed to determine mean parameters and SD.

### Western blotting

Cells were collected by spinning down trypsinized cultures at ∼200 *g* for 5 min at 4°C; trypsin was neutralized by addition of FBS before centrifugation. Cell pellets were washed with ice-cold PBS and lysed in ice-cold radioimmunoprecipitation assay buffer supplemented with Halt Protease and Phosphatase Inhibitor Cocktail (Thermo Fisher Scientific) for 30 min. Lysates were cleared by centrifugation at 16,000 *g* for 10 min at 4°C and boiled in NuPAGE protein sample buffer (Thermo Fisher Scientific) containing 5% 2-mercaptoethanol (Sigma-Aldrich). Protein samples were separated by SDS-PAGE in 4–12% Bis-Tris gels (Thermo Fisher Scientific), transferred to polyvinylidene difluoride membrane, blocked in SuperBlock (TBS) Blocking Buffer (Thermo Fisher Scientific), and washed with TBST. Primary antibodies were detected using HRP-conjugated secondary antibodies and developed using the ECL 2 (Pierce) or WesternBright (Advansta) detection kit. Chemiluminescence was detected using G:Box Chemi XT4 (Syngene).

### Antibodies

Antibodies used in this study are as follows: UBF (F-9) mouse monoclonal antibody used for immuno-FISH (sc-13125; Santa Cruz Biotechnology), UBF (M01) mouse monoclonal antibody used for immunofluorescence and Western blotting (clone 6B6; Abnova), c-Myc rabbit polyclonal antibody (5605; Cell Signaling Technology), nucleolin rabbit polyclonal antibody (ab70493; Abcam), GAPDH (D16H11) rabbit mAb (5174; Cell Signaling Technology), RAD21 (D213) antibody (4321; Cell Signaling Technology), β-Actin (8H10D10) mouse mAb (3700; Cell Signaling Technology), Ki-67 (8D5) mouse mAb (9449; Cell Signaling Technology), and α-tubulin antibody (ab15246; 1:500; Abcam). Secondary antibodies for immunofluorescence (Alexa Fluor 488 and 594 conjugates) were obtained from Life Technologies and used at 1:500 dilution. Secondary HRP-conjugated antibodies for Western blotting were from Cell Signaling Technology and typically used at 1:5,000 dilution.

### Data availability

Original data underlying this article can be accessed from the Stowers Original Data Repository at https://www.stowers.org/research/publications/libpb-1430.

### Online supplemental material

Figs. S1–S5 show validation of rDNA linkages by SIM and STED super-resolution microscopy, telomere and rDNA FISH, chromosomal spreads treated with RNAse, rDNA linkages between mouse chromosomes, cytogenetic and genomic characterizations of c-Myc overexpressing RPE1 clonal derivatives, UBF negative rDNA loci in CHON tetraploid-2, rDNA linkages in Rad21 knockdown, and lack of rDNA linkage resolution in cells treated with Dexrazoxane. Videos 1–3 show mitosis in RPE1 cells expressing eGFP-UBF: untreated cells and cells treated with Topoisomerase inhibitors ICRF-193 or Dexrazoxane. Tables S1–S3 show a summary of chromosomal spread data from mouse–human hybrid cell line GM15292 and diploid CHON-002 cell line and its tetraploid derivatives, CHON tetraploid 1 and CHON tetraploid 2.

## Supplementary Material

Supplemental Materials

Tables S1--S3 (zipped Excel files)

Video 1

Video 2

Video 3

## References

[bib1] Ardito G., Sarti-Chiarelli M., and De Boer L.E.M. 1973 Association between Acrocentric Chromosomes in the Chimpanzee (Pan Troglodytes). Boll. Zool. 40:361–366.

[bib2] Ardito G., Lamberti L., and Brøgger A. 1978 Satellite associations of human acrocentric chromosomes identified by trypsin treatment at metaphase. Ann. Hum. Genet. 41:455–462. 10.1111/j.1469-1809.1978.tb00915.x 655634

[bib3] Ball G., Demmerle J., Kaufmann R., Davis I., Dobbie I.M., and Schermelleh L. 2015 SIMcheck: a Toolbox for Successful Super-resolution Structured Illumination Microscopy. Sci. Rep. 5:15915 10.1038/srep15915 26525406 PMC4648340

[bib4] Bauer D.L., Marie R., Rasmussen K.H., Kristensen A., and Mir K.U. 2012 DNA catenation maintains structure of human metaphase chromosomes. Nucleic Acids Res. 40:11428–11434. 10.1093/nar/gks931 23066100 PMC3526300

[bib5] Bolzer A., Kreth G., Solovei I., Koehler D., Saracoglu K., Fauth C., Müller S., Eils R., Cremer C., Speicher M.R., and Cremer T. 2005 Three-dimensional maps of all chromosomes in human male fibroblast nuclei and prometaphase rosettes. PLoS Biol. 3:e157 10.1371/journal.pbio.0030157 15839726 PMC1084335

[bib6] Caradonna F. 2015 Nucleoplasmic bridges and acrocentric chromosome associations as early markers of exposure to low levels of ionising radiation in occupationally exposed hospital workers. Mutagenesis. 30:269–275. 10.1093/mutage/geu068 25381312

[bib7] Cazaux B., Catalan J., Veyrunes F., Douzery E.J., and Britton-Davidian J. 2011 Are ribosomal DNA clusters rearrangement hotspots?: a case study in the genus Mus (Rodentia, Muridae). BMC Evol. Biol. 11:124 10.1186/1471-2148-11-124 21569527 PMC3112088

[bib8] Chen D., and Huang S. 2001 Nucleolar components involved in ribosome biogenesis cycle between the nucleolus and nucleoplasm in interphase cells. J. Cell Biol. 153:169–176. 10.1083/jcb.153.1.169 11285283 PMC2185520

[bib9] Chen D., Dundr M., Wang C., Leung A., Lamond A., Misteli T., and Huang S. 2005 Condensed mitotic chromatin is accessible to transcription factors and chromatin structural proteins. J. Cell Biol. 168:41–54. 10.1083/jcb.200407182 15623580 PMC2171683

[bib10] Cremer T., Kurz A., Zirbel R., Dietzel S., Rinke B., Schröck E., Speicher M.R., Mathieu U., Jauch A., Emmerich P., 1993 Role of chromosome territories in the functional compartmentalization of the cell nucleus. Cold Spring Harb. Symp. Quant. Biol. 58:777–792. 10.1101/SQB.1993.058.01.085 7525149

[bib11] Daum J.R., Mo Y.Y., and Gorbsky G.J. 2009 The dynamics of DNA topoisomerase IIalpha in living cells. Methods Mol. Biol. 582:233–244. 10.1007/978-1-60761-340-4_18 19763954 PMC2950002

[bib12] Dundr M., Hoffmann-Rohrer U., Hu Q., Grummt I., Rothblum L.I., Phair R.D., and Misteli T. 2002 A kinetic framework for a mammalian RNA polymerase in vivo. Science. 298:1623–1626. 10.1126/science.1076164 12446911

[bib13] Ferguson-Smith M.A., and Handmaker S.D. 1961 Observations on the satellited human chromosomes. Lancet. 1:638–640. 10.1016/S0140-6736(61)91655-5 13698902

[bib14] Floutsakou I., Agrawal S., Nguyen T.T., Seoighe C., Ganley A.R., and McStay B. 2013 The shared genomic architecture of human nucleolar organizer regions. Genome Res. 23:2003–2012. 10.1101/gr.157941.113 23990606 PMC3847771

[bib15] Frolov A.K., Sokhin A.A., Frolov V.K., and Lebedinskii A.P. 1975 [Frequency of acrocentric chromosomal associations in children immunized with smallpox vaccine]. Tsitologiia. 17:1177–1183. 1189038

[bib16] Garbe J.C., Bhattacharya S., Merchant B., Bassett E., Swisshelm K., Feiler H.S., Wyrobek A.J., and Stampfer M.R. 2009 Molecular distinctions between stasis and telomere attrition senescence barriers shown by long-term culture of normal human mammary epithelial cells. Cancer Res. 69:7557–7568. 10.1158/0008-5472.CAN-09-0270 19773443 PMC2782785

[bib17] Garbe J.C., Vrba L., Sputova K., Fuchs L., Novak P., Brothman A.R., Jackson M., Chin K., LaBarge M.A., Watts G., 2014 Immortalization of normal human mammary epithelial cells in two steps by direct targeting of senescence barriers does not require gross genomic alterations. Cell Cycle. 13:3423–3435. 10.4161/15384101.2014.954456 25485586 PMC4613853

[bib18] Gibbons J.G., Branco A.T., Godinho S.A., Yu S., and Lemos B. 2015 Concerted copy number variation balances ribosomal DNA dosage in human and mouse genomes. Proc. Natl. Acad. Sci. USA. 112:2485–2490. 10.1073/pnas.1416878112 25583482 PMC4345604

[bib19] Gorbsky G.J. 1994 Cell cycle progression and chromosome segregation in mammalian cells cultured in the presence of the topoisomerase II inhibitors ICRF-187 [(+)-1,2-bis(3,5-dioxopiperazinyl-1-yl)propane; ADR-529] and ICRF-159 (Razoxane). Cancer Res. 54:1042–1048. 8313360

[bib20] Grandori C., Gomez-Roman N., Felton-Edkins Z.A., Ngouenet C., Galloway D.A., Eisenman R.N., and White R.J. 2005 c-Myc binds to human ribosomal DNA and stimulates transcription of rRNA genes by RNA polymerase I. Nat. Cell Biol. 7:311–318. 10.1038/ncb1224 15723054

[bib21] Grummt I., and Pikaard C.S. 2003 Epigenetic silencing of RNA polymerase I transcription. Nat. Rev. Mol. Cell Biol. 4:641–649. 10.1038/nrm1171 12923526

[bib22] Gustafsson M.G. 2000 Surpassing the lateral resolution limit by a factor of two using structured illumination microscopy. J. Microsc. 198:82–87. 10.1046/j.1365-2818.2000.00710.x 10810003

[bib23] Haarhuis J.H., Elbatsh A.M., and Rowland B.D. 2014 Cohesin and its regulation: on the logic of X-shaped chromosomes. Dev. Cell. 31:7–18. 10.1016/j.devcel.2014.09.010 25313959

[bib24] Hansson A. 1979 Satellite association in human metaphases. A comparative study of normal individuals, patients with Down syndrome and their parents. Hereditas. 90:59–83. 10.1111/j.1601-5223.1979.tb01294.x 154489

[bib25] Heintzmann R., and Huser T. 2017 Super-Resolution Structured Illumination Microscopy. Chem. Rev. 117:13890–13908. 10.1021/acs.chemrev.7b00218 29125755

[bib26] Hell S.W., and Wichmann J. 1994 Breaking the diffraction resolution limit by stimulated emission: stimulated-emission-depletion fluorescence microscopy. Opt. Lett. 19:780–782. 10.1364/OL.19.000780 19844443

[bib27] Henderson A.S., Warburton D., and Atwood K.C. 1972 Location of ribosomal DNA in the human chromosome complement. Proc. Natl. Acad. Sci. USA. 69:3394–3398. 10.1073/pnas.69.11.3394 4508329 PMC389778

[bib28] Herdman C., Mars J.C., Stefanovsky V.Y., Tremblay M.G., Sabourin-Felix M., Lindsay H., Robinson M.D., and Moss T. 2017 A unique enhancer boundary complex on the mouse ribosomal RNA genes persists after loss of Rrn3 or UBF and the inactivation of RNA polymerase I transcription. PLoS Genet. 13:e1006899 10.1371/journal.pgen.1006899 28715449 PMC5536353

[bib29] Ishida R., Sato M., Narita T., Utsumi K.R., Nishimoto T., Morita T., Nagata H., and Andoh T. 1994 Inhibition of DNA topoisomerase II by ICRF-193 induces polyploidization by uncoupling chromosome dynamics from other cell cycle events. J. Cell Biol. 126:1341–1351. 10.1083/jcb.126.6.1341 8089169 PMC2290951

[bib30] Jacobs P.A., Mayer M., and Morton N.E. 1976 Acrocentric chromosome associations in man. Am. J. Hum. Genet. 28:567–576. 795295 PMC1685176

[bib31] Jao C.Y., and Salic A. 2008 Exploring RNA transcription and turnover in vivo by using click chemistry. Proc. Natl. Acad. Sci. USA. 105:15779–15784. 10.1073/pnas.0808480105 18840688 PMC2572917

[bib32] Kim L.K., Esplugues E., Zorca C.E., Parisi F., Kluger Y., Kim T.H., Galjart N.J., and Flavell R.A. 2014 Oct-1 regulates IL-17 expression by directing interchromosomal associations in conjunction with CTCF in T cells. Mol. Cell. 54:56–66. 10.1016/j.molcel.2014.02.004 24613343 PMC4058095

[bib33] Lee J.K., Garbe J.C., Vrba L., Miyano M., Futscher B.W., Stampfer M.R., and LaBarge M.A. 2015 Age and the means of bypassing stasis influence the intrinsic subtype of immortalized human mammary epithelial cells. Front. Cell Dev. Biol. 3:13 10.3389/fcell.2015.00013 25815289 PMC4356162

[bib34] Lezhava T.A. 1979 Human acrocentric chromosomal associations in old age. Tsitol. Genet. 13:481–485. 552667

[bib35] Livak K.J., and Schmittgen T.D. 2001 Analysis of relative gene expression data using real-time quantitative PCR and the 2(-Delta Delta C(T)) Method. Methods. 25:402–408. 10.1006/meth.2001.1262 11846609

[bib36] Maass P.G., Barutcu A.R., Weiner C.L., and Rinn J.L. 2018 Inter-chromosomal Contact Properties in Live-Cell Imaging and in Hi-C. Mol. Cell. 70:188–189. 10.1016/j.molcel.2018.03.021 29625035 PMC5896573

[bib37] Mais C., Wright J.E., Prieto J.L., Raggett S.L., and McStay B. 2005 UBF-binding site arrays form pseudo-NORs and sequester the RNA polymerase I transcription machinery. Genes Dev. 19:50–64. 10.1101/gad.310705 15598984 PMC540225

[bib38] Maniotis A.J., Bojanowski K., and Ingber D.E. 1997 Mechanical continuity and reversible chromosome disassembly within intact genomes removed from living cells. J. Cell. Biochem. 65:114–130. 10.1002/(SICI)1097-4644(199704)65:1<114::AID-JCB12>3.0.CO;2-K 9138086

[bib39] Marko J.F. 2008 Micromechanical studies of mitotic chromosomes. Chromosome Res. 16:469–497. 10.1007/s10577-008-1233-7 18461485

[bib40] Mars J.C., Sabourin-Felix M., Tremblay M.G., and Moss T. 2018 A Deconvolution Protocol for ChIP-Seq Reveals Analogous Enhancer Structures on the Mouse and Human Ribosomal RNA Genes. G3 (Bethesda). 8:303–314. 10.1534/g3.117.300225 29158335 PMC5765358

[bib41] McDowell K.A., Hadjiargyrou M., Patsalis P., and Henderson A.S. 1994 Transcription of rDNA is essential for satellite association. Cytogenet. Cell Genet. 66:63–67. 10.1159/000133666 8275712

[bib42] McKinlay Gardner R.J., Sutherland G.R., Shaffer L.G., eds. 2011 Chromosome Abnormalities and Genetic Counseling. Oxford University Press, New York.

[bib43] McStay B. 2016 Nucleolar organizer regions: genomic ‘dark matter’ requiring illumination. Genes Dev. 30:1598–1610. 10.1101/gad.283838.116 27474438 PMC4973289

[bib44] McStay B., and Grummt I. 2008 The epigenetics of rRNA genes: from molecular to chromosome biology. Annu. Rev. Cell Dev. Biol. 24:131–157. 10.1146/annurev.cellbio.24.110707.175259 18616426

[bib45] Meaburn K.J., and Misteli T. 2007 Cell biology: chromosome territories. Nature. 445:379–381. 10.1038/445379a 17251970

[bib46] Müller T., Schumann C., and Kraegeloh A. 2012 STED microscopy and its applications: new insights into cellular processes on the nanoscale. ChemPhysChem. 13:1986–2000. 10.1002/cphc.201100986 22374829

[bib47] Nitiss J.L. 2009 DNA topoisomerase II and its growing repertoire of biological functions. Nat. Rev. Cancer. 9:327–337. 10.1038/nrc2608 19377505 PMC2730144

[bib48] O’Sullivan A.C., Sullivan G.J., and McStay B. 2002 UBF binding in vivo is not restricted to regulatory sequences within the vertebrate ribosomal DNA repeat. Mol. Cell. Biol. 22:657–668. 10.1128/MCB.22.2.657-668.2002 11756560 PMC139743

[bib49] Palazzo A.F., and Lee E.S. 2015 Non-coding RNA: what is functional and what is junk? Front. Genet. 6:2 10.3389/fgene.2015.00002 25674102 PMC4306305

[bib50] Poirier M.G., and Marko J.F. 2003 Micromechanical studies of mitotic chromosomes. Curr. Top. Dev. Biol. 55:75–141. 10.1016/S0070-2153(03)01002-0 12959194

[bib51] Pommier Y., Sun Y., Huang S.N., and Nitiss J.L. 2016 Roles of eukaryotic topoisomerases in transcription, replication and genomic stability. Nat. Rev. Mol. Cell Biol. 17:703–721. 10.1038/nrm.2016.111 27649880 PMC9248348

[bib52] Potapova T.A., Seidel C.W., Box A.C., Rancati G., and Li R. 2016 Transcriptome analysis of tetraploid cells identifies cyclin D2 as a facilitator of adaptation to genome doubling in the presence of p53. Mol. Biol. Cell. 27:3065–3084. 10.1091/mbc.e16-05-0268 27559130 PMC5063615

[bib53] Roussel P., André C., Masson C., Géraud G., and Hernandez-Verdun D. 1993 Localization of the RNA polymerase I transcription factor hUBF during the cell cycle. J. Cell Sci. 104:327–337. 8505363 10.1242/jcs.104.2.327

[bib54] Sanij E., and Hannan R.D. 2009 The role of UBF in regulating the structure and dynamics of transcriptionally active rDNA chromatin. Epigenetics. 4:374–382. 10.4161/epi.4.6.9449 19717978

[bib55] Sanij E., Poortinga G., Sharkey K., Hung S., Holloway T.P., Quin J., Robb E., Wong L.H., Thomas W.G., Stefanovsky V., 2008 UBF levels determine the number of active ribosomal RNA genes in mammals. J. Cell Biol. 183:1259–1274. 10.1083/jcb.200805146 19103806 PMC2606969

[bib56] Scherer S. 2008 A Short Guide to the Human Genome. Cold Spring Harbor Laboratory Press, Cold Spring Harbor, NY.

[bib57] Sen N., Leonard J., Torres R., Garcia-Luis J., Palou-Marin G., and Aragón L. 2016 Physical Proximity of Sister Chromatids Promotes Top2-Dependent Intertwining. Mol. Cell. 64:134–147. 10.1016/j.molcel.2016.09.007 27716481 PMC5065527

[bib58] Slaughter B.D., Unruh J.R., Das A., Smith S.E., Rubinstein B., and Li R. 2013 Non-uniform membrane diffusion enables steady-state cell polarization via vesicular trafficking. Nat. Commun. 4:1380 10.1038/ncomms2370 23340420 PMC3900288

[bib59] Spilianakis C.G., and Flavell R.A. 2004 Long-range intrachromosomal interactions in the T helper type 2 cytokine locus. Nat. Immunol. 5:1017–1027. 10.1038/ni1115 15378057

[bib60] Stampfer M.R., and Bartley J.C. 1985 Induction of transformation and continuous cell lines from normal human mammary epithelial cells after exposure to benzo[a]pyrene. Proc. Natl. Acad. Sci. USA. 82:2394–2398. 10.1073/pnas.82.8.2394 3857588 PMC397564

[bib61] Stampfer M.R., Garbe J., Levine G., Lichtsteiner S., Vasserot A.P., and Yaswen P. 2001 Expression of the telomerase catalytic subunit, hTERT, induces resistance to transforming growth factor beta growth inhibition in p16INK4A(-) human mammary epithelial cells. Proc. Natl. Acad. Sci. USA. 98:4498–4503. 10.1073/pnas.071483998 11287649 PMC31863

[bib62] Stampfer M.R., Garbe J., Nijjar T., Wigington D., Swisshelm K., and Yaswen P. 2003 Loss of p53 function accelerates acquisition of telomerase activity in indefinite lifespan human mammary epithelial cell lines. Oncogene. 22:5238–5251. 10.1038/sj.onc.1206667 12917625

[bib63] Stevens J.B., Abdallah B.Y., Regan S.M., Liu G., Bremer S.W., Ye C.J., and Heng H.H. 2010 Comparison of mitotic cell death by chromosome fragmentation to premature chromosome condensation. Mol. Cytogenet. 3:20 10.1186/1755-8166-3-20 20959006 PMC2974731

[bib64] Stimpson K.M., Sullivan L.L., Kuo M.E., and Sullivan B.A. 2014 Nucleolar organization, ribosomal DNA array stability, and acrocentric chromosome integrity are linked to telomere function. PLoS One. 9:e92432 10.1371/journal.pone.0092432 24662969 PMC3963894

[bib65] Tajrishi M.M., Tuteja R., and Tuteja N. 2011 Nucleolin: The most abundant multifunctional phosphoprotein of nucleolus. Commun. Integr. Biol. 4:267–275. 10.4161/cib.4.3.14884 21980556 PMC3187884

[bib66] Tucker S., Vitins A., and Pikaard C.S. 2010 Nucleolar dominance and ribosomal RNA gene silencing. Curr. Opin. Cell Biol. 22:351–356. 10.1016/j.ceb.2010.03.009 20392622 PMC2912983

[bib67] Uetake Y., and Sluder G. 2010 Prolonged prometaphase blocks daughter cell proliferation despite normal completion of mitosis. Curr. Biol. 20:1666–1671. 10.1016/j.cub.2010.08.018 20832310 PMC2946429

[bib68] Uhlmann F., Lottspeich F., and Nasmyth K. 1999 Sister-chromatid separation at anaphase onset is promoted by cleavage of the cohesin subunit Scc1. Nature. 400:37–42. 10.1038/21831 10403247

[bib69] van Riggelen J., Yetil A., and Felsher D.W. 2010 MYC as a regulator of ribosome biogenesis and protein synthesis. Nat. Rev. Cancer. 10:301–309. 10.1038/nrc2819 20332779

[bib70] Vassilev L.T., Tovar C., Chen S., Knezevic D., Zhao X., Sun H., Heimbrook D.C., and Chen L. 2006 Selective small-molecule inhibitor reveals critical mitotic functions of human CDK1. Proc. Natl. Acad. Sci. USA. 103:10660–10665. 10.1073/pnas.0600447103 16818887 PMC1502288

[bib71] Vortkamp A., Thias U., Gessler M., Rosenkranz W., Kroisel P.M., Tommerup N., Krüger G., Götz J., Pelz L., and Grzeschik K.H. 1991 A somatic cell hybrid panel and DNA probes for physical mapping of human chromosome 7p. Genomics. 11:737–743. 10.1016/0888-7543(91)90082-P 1663489

[bib72] Watanabe-Susaki K., Takada H., Enomoto K., Miwata K., Ishimine H., Intoh A., Ohtaka M., Nakanishi M., Sugino H., Asashima M., and Kurisaki A. 2014 Biosynthesis of ribosomal RNA in nucleoli regulates pluripotency and differentiation ability of pluripotent stem cells. Stem Cells. 32:3099–3111. 10.1002/stem.1825 25187421

[bib73] Williams A., Spilianakis C.G., and Flavell R.A. 2010 Interchromosomal association and gene regulation in trans. Trends Genet. 26:188–197. 10.1016/j.tig.2010.01.007 20236724 PMC2865229

[bib74] Wren M.C., Zhao J., Liu C.C., Murray M.E., Atagi Y., Davis M.D., Fu Y., Okano H.J., Ogaki K., Strongosky A.J., 2015 Frontotemporal dementia-associated N279K tau mutant disrupts subcellular vesicle trafficking and induces cellular stress in iPSC-derived neural stem cells. Mol. Neurodegener. 10:46 10.1186/s13024-015-0042-7 26373282 PMC4572645

[bib75] Xu B., Li H., Perry J.M., Singh V.P., Unruh J., Yu Z., Zakari M., McDowell W., Li L., and Gerton J.L. 2017 a Ribosomal DNA copy number loss and sequence variation in cancer. PLoS Genet. 13:e1006771 10.1371/journal.pgen.1006771 28640831 PMC5480814

[bib76] Xu J., Peng X., Chen Y., Zhang Y., Ma Q., Liang L., Carter A.C., Lu X., and Wu C.I. 2017 b Free-living human cells reconfigure their chromosomes in the evolution back to uni-cellularity. eLife. 6:e28070 10.7554/eLife.28070 29251591 PMC5734875

[bib77] Yasseen A.A., and Al-Musawi T.A. 2001 Metaphase acrocentric associations in mentally retarded patients. Neurosciences (Riyadh). 6:233–237. 24185186

[bib78] Zentner G.E., Saiakhova A., Manaenkov P., Adams M.D., and Scacheri P.C. 2011 Integrative genomic analysis of human ribosomal DNA. Nucleic Acids Res. 39:4949–4960. 10.1093/nar/gkq1326 21355038 PMC3130253

[bib79] Zhdanova N.S. 1972 [Acrocentric chromosome associations in human lymphocytes]. Tsitologiia. 14:1098–1205. 4265915

