# Supplementary material for: Superresolution microscopy reveals linkages between ribosomal DNA on heterologous chromosomes
Source: J Cell Biol. 2019 Jul 3;218(8):2492–513. doi: 10.1083/jcb.201810166 (PMC6683752; doi:10.1083/jcb.201810166)
Supplement: Supplemental Materials [file JCB_201810166_sm.pdf]

JCB

Supplemental material

Potapova et al., https://doi.org/10.1083/jcb.201810166

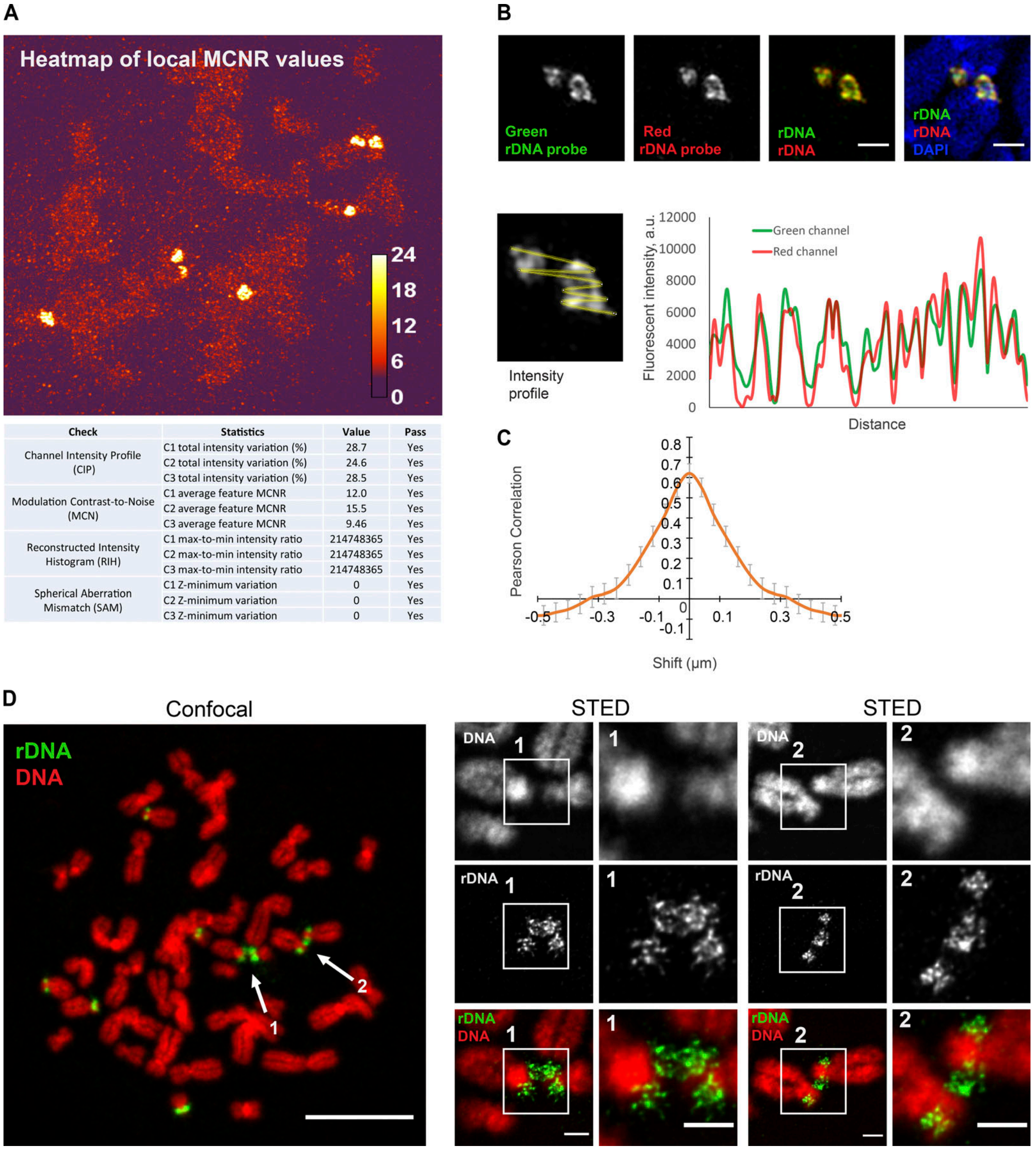

Check	Statistics	Value	Pass
Channel Intensity Profile (CIP)	C1 total intensity variation (%)	28.7	Yes
	C2 total intensity variation (%)	24.6	Yes
	C3 total intensity variation (%)	28.5	Yes
Modulation Contrast-to-Noise (MCN)	C1 average feature MCNR	12.0	Yes
	C2 average feature MCNR	15.5	Yes
	C3 average feature MCNR	9.46	Yes
Reconstructed Intensity Histogram (RIH)	C1 max-to-min intensity ratio	214748365	Yes
	C2 max-to-min intensity ratio	214748365	Yes
	C3 max-to-min intensity ratio	214748365	Yes
Spherical Aberration Mismatch (SAM)	C1 Z-minimum variation	0	Yes
	C2 Z-minimum variation	0	Yes
	C3 Z-minimum variation	0	Yes

Figure S1. **Validation of rDNA linkages by SIM and STED superresolution microscopy. (A)** SIMcheck analysis of the data quality for images shown in Fig. 1, B and C. Top panel shows the heatmap of local modulation contrast-to-noise ratio (MCNR) values. Color of a lookup table (LUT) is indicative of the MCNR value. Orange (MCNR = 8–12) indicates good MCN ratio, and yellow-white (MCNR >12) indicates very good to excellent MCN ratio. A table below summarizes the output of SIMcheck criteria. For details on SIMcheck analysis, refer to Ball et al. (2015). **(B)** SIM images of linked mitotic acrocentric chromosomes labeled by FISH with rDNA probes labeled with green (FITC) and red (5-ROX) dUTPs. Both probes were generated from the same BAC clone, RP11-450E20. The same rDNA linkage structure is seen in both green and red channels, making it unlikely that this structure is an artifact of SIM image reconstruction. The graph below shows the intensity profile of the segmented line drawn through the rDNA loci. While peak intensity values in green and red channels are not precisely equivalent, signals from both channels overlap spatially (colocalize). Bar, 1 µm. **(C)** Spatial Pearson cross-correlation profile between green and red fluorescent signal of the mitotic chromosome spreads labeled by FISH with green (5-fluorescein) and red (5-ROX) rDNA probes as in B. Multiple line profiles were drawn through several rDNA loci, and the Pearson correlation coefficient was measured as a function of shifting the channels relative to each other. The peak at the 0 shift value indicates the fraction of colocalized signal. The conical shape of the peak indicates that processed SIM images are free of reconstruction artifacts that tend to display periodic patterns. **(D)** Confocal and STED images of mitotic chromosome spread from hTERT RPE1 cell labeled by FISH with rDNA probe (green). Chromosomes are shown in red. Arrows 1 and 2 on the confocal image point to acrocentric chromosomal rDNA associations. Bar, 10 µm. Panels on the right show deconvolved STED images of connected chromosomes with magnified rDNA linkages. Images of rDNA linkages obtained by SIM and STED superresolution methods show very similar structures. Bar, 1 µm.

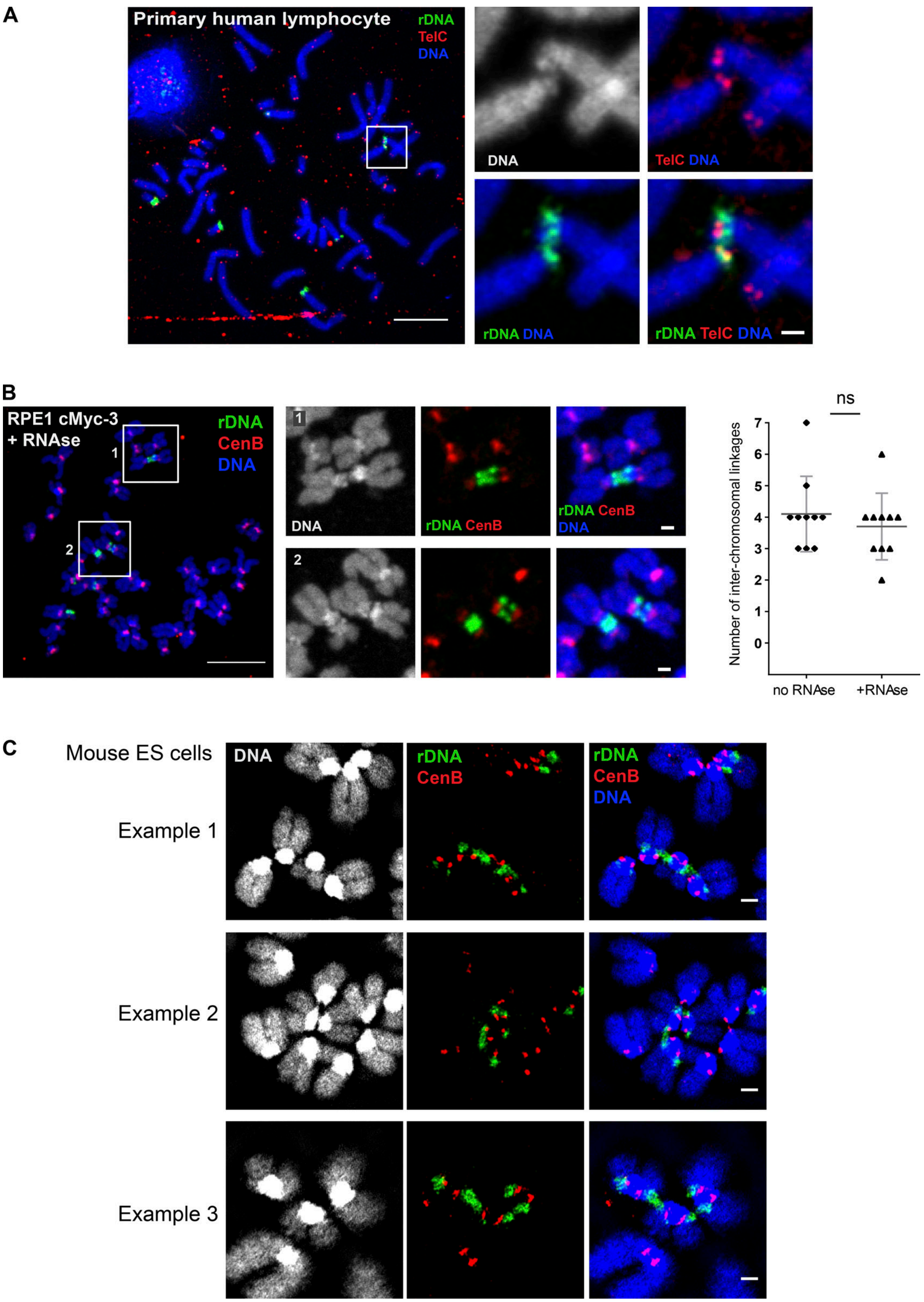

Figure S2. **Telomeres and rDNA linkages, effect of RNAse treatment, and mouse rDNA linkages. (A)** Telomere (red) and rDNA (green) FISH of mitotic chromosome spreads from primary human lymphocytes. Bar, 10 µm. Inset shows a magnified pair of acrocentric chromosomes connected laterally by an rDNA bridge. Their short-arm telomeres are intact and not fused. Bar, 1 µm. **(B)** Representative chromosomal spread from c-Myc–overexpressing derivative cMyc-3 treated with 1 mg/ml of RNAse A during the hypotonic swelling step of spread preparation. Spreads were labeled by FISH with rDNA probe (green) and CenB probe (red). Bar, 10 µm. Panels on the right show persistent acrocentric associations. Bar, 1 µm. The graph on the left shows quantification of interchromosomal rDNA linkages in cMyc-3 cell line with and without RNAse treatment. 10 chromosomal spreads from each cell line were examined; no significant (ns) difference was detected. **(C)** Representative SIM images of rDNA linkages between mouse chromosomes from the V6.5 mouse embryonic stem cell line. Chromosomal spreads were labeled by FISH with rDNA probe (green) and CenB probe (red). Bar, 1 µm.

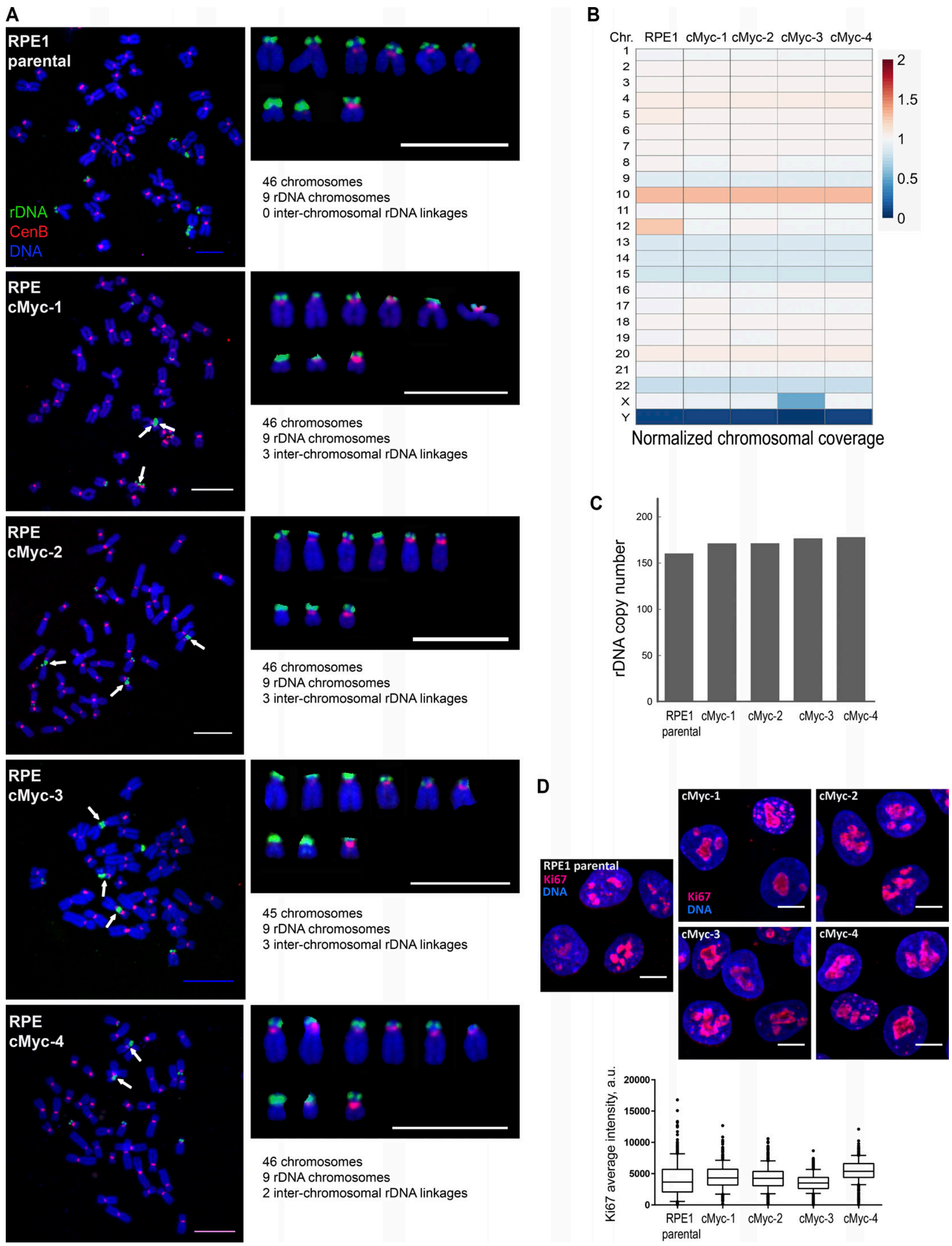

Figure S3. **Additional cytogenetic and genomic characterizations of c-Myc–overexpressing clonal derivatives (cMyc 1–4) of RPE1 cell line. (A)** Representative chromosomal spreads from parental RPE1 cell line and c-Myc–overexpressing single cell clones 1–4 labeled by FISH with rDNA probe (green) and CenB probe (red). Panels on the right show individual large and small acrocentric chromosomes. Individual chromosomes containing rDNA were manually segmented, rotated, and arranged according to their size. Parental and c-Myc derivatives of RPE1 are all lacking rDNA on one of the small acrocentric chromosomes (likely chromosome 22). Arrows point to interchromosomal rDNA linkages. Bar, 10 µm. **(B)** Next-generation DNA-seq analysis of chromosome (Chr.) copy number. The scale reflects normalized chromosomal coverage for each cell line. All cell lines are diploid with the following small abnormalities: All show a small gain of chromosome 10, which reflects a known karyotypic abnormality in the RPE1 cell line where an additional piece of chromosome 10 is translocated on the q arm of chromosome X (Potapova et al., 2015); parental RPE1 shows a gain of chromosome 12, reflecting the tendency of the RPE1 cell line to become trisomic for chromosome 12 with passaging (Potapova et al., 2016); the clone RPE cMyc-3 lost a copy of chromosome X. **(C)** 45S copy number in the c-Myc–overexpressing single-cell clones was calculated using coverage from DNA-seq, using a computational method described in Xu et al. (2017). Copy number in all clones was similar to the parental RPE1 cell line. **(D)** Representative spinning disk confocal images of Ki67 immunofluorescence (red) of parental RPE1 cells and c-Myc–overexpressing derivatives. Nuclei were counterstained with DAPI (blue). Bar, 10 µm. The box plot shows quantification of the average fluorescent intensity of Ki67 per nucleus. Three montage images (2 × 2 fields) containing tens to hundreds of cells were quantified per cell line. The average intensity of Ki67 is in the same range in parental and c-Myc–overexpressing cell lines.

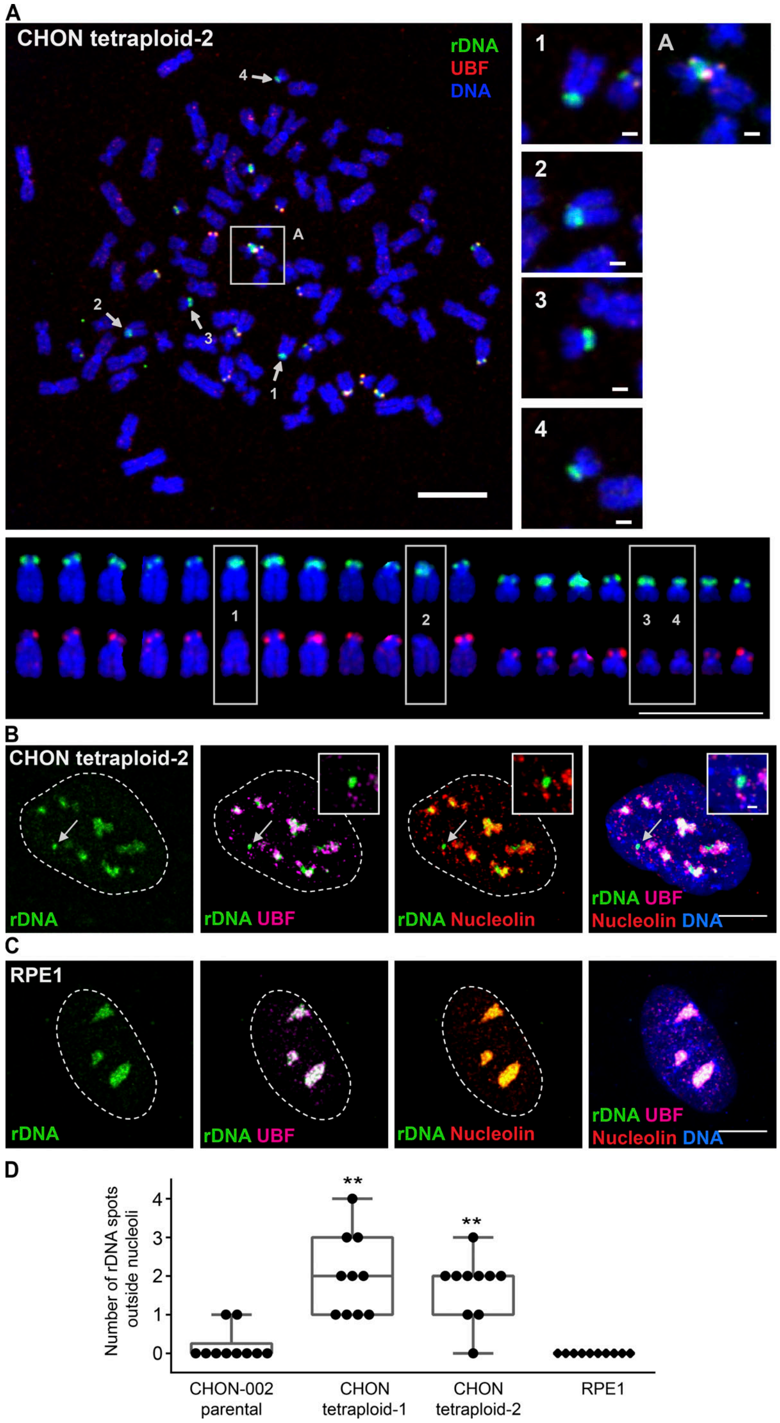

Figure S4. **Tetraploid derivative CHON tetraploid-2 display some UBF-negative rDNA loci that do not form rDNA linkages and are not incorporated in nucleoli.** Chromosome spread from CHON tetraploid-1 cell labeled by immuno-FISH with rDNA probe (green) and UBF antibody (red). **(A)** Boxes highlight rDNA linkages that were formed between UBF⁺ rDNA chromosomes (magnified on the right). Arrows 1–4 point to rDNA chromosomes lacking UBF. Panels below show individual rDNA chromosomes labeled with rDNA probe (top row) and UBF antibody (bottom row). Individual chromosomes containing rDNA were manually segmented, rotated, and arranged according to their size. In the spread shown, there were 20 rDNA chromosomes, 16 of which were UBF^+ and four were UBF^- (boxes 1–4, magnified on the right). Bar, 10 µm; magnified insets, 1 µm. **(B and C)** Interphase nuclei of tetraploid CHON tetraploid-2 cell line (B) and RPE1 cell line (C). Cells were labeled by immuno-FISH with rDNA probe (green), nucleolin antibody (red), and UBF antibody (magenta). In the tetraploid nucleus (B), a compact rDNA spot is present that is UBF negative and nucleolin negative (arrow, magnified inset). In the RPE1 nucleus (C), all rDNA is associated with UBF and nucleolin. Bar, 10 µm; magnified inset, 1 µm. **(D)** Quantification of rDNA loci not incorporated in nucleoli. The number of rDNA spots that were completely outside the nucleoli was counted in 10 nuclei from each cell line. The number of rDNA loci nonoverlapping with nucleolin was significantly higher in both tetraploid cell lines compared to the parental diploid CHON-002 cell line (*t* test: **, $P < 0.001$).

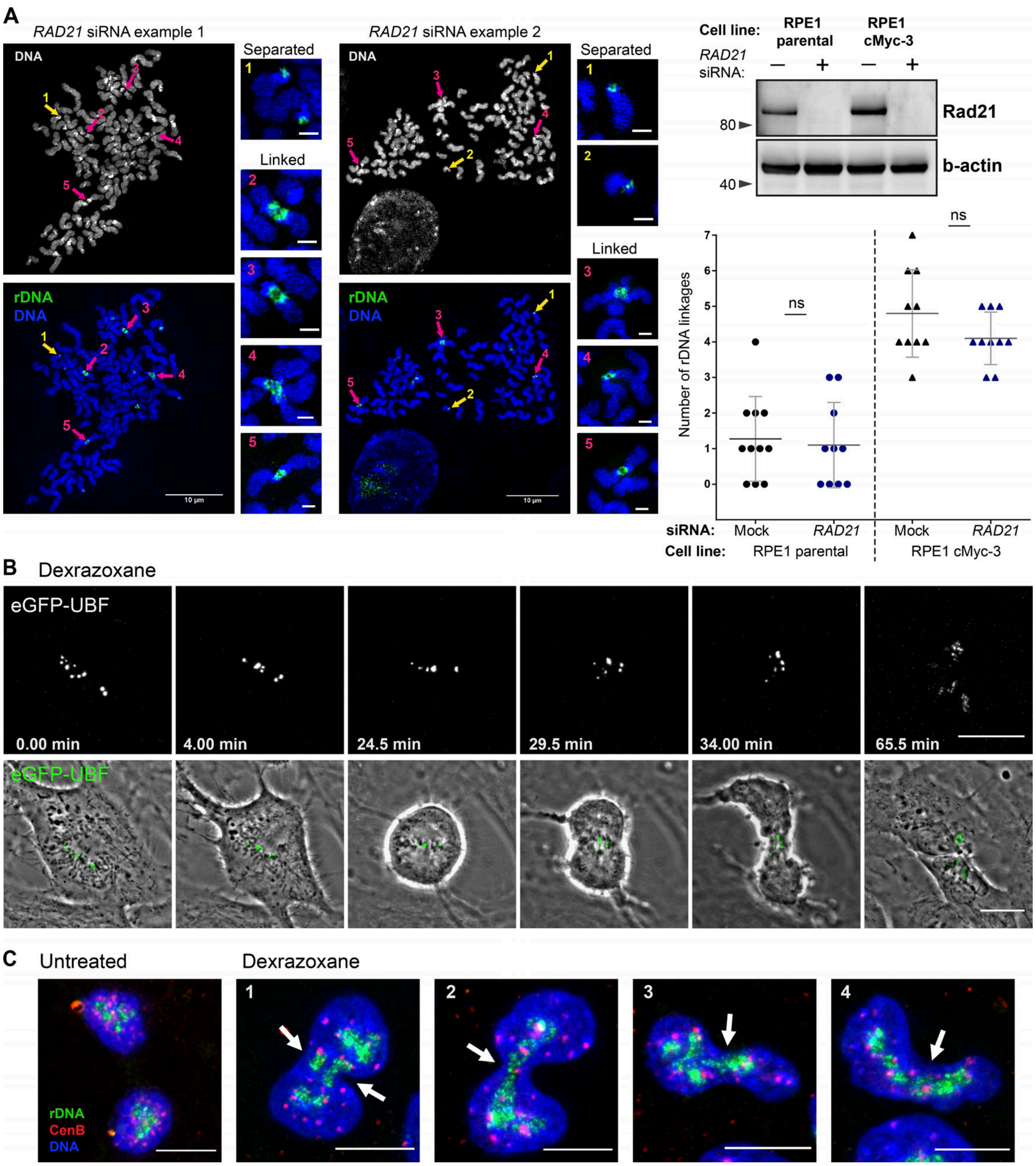

Figure S5. **Persistence of rDNA linkages in the absence of cohesin, and impaired rDNA segregation in the presence of Topoisomerase II inhibitor dexrazoxane. (A)** Loss of cohesin does not lead to resolution of rDNA linkages in metaphase. The left panel shows chromosome spreads from c-Myc–overexpressing RPE1 derivative cell line cMyc-3 treated with siRNA to cohesin subunit Rad21 for 72 h and labeled by FISH with a probe to the rDNA (green). Without Rad21, sister chromatids separate in mitotically arrested cells. However, rDNA linkages connecting sister chromatids or different chromosomes persist. Yellow arrows point to separated (nonlinked) rDNA loci. Red arrows point to rDNA linkages connecting otherwise separated chromatids. Bar, 10 µm; magnified insets, 1 µm. Lateral panels show magnified views of these rDNA loci. Two examples of the same phenotype are shown. The left panel shows Western blot analysis and quantification of the number of rDNA linkages in parental RPE1 cells and c-Myc–overexpressing derivative cell line cMyc-3 transfected with *RAD21* siRNA. *RAD21* siRNA induced strong protein knockdown. Knockdown of *RAD21* does not cause a significant decrease in the frequency of rDNA linkages in either cell line. Images of ≥10 chromosomal spreads from each sample were examined. The difference between the mock and *RAD21* siRNA for each cell line was evaluated using Mann–Whitney *U* test. ns, not significant. **(B)** Topoisomerase II inhibitor dexrazoxane prevents resolution of rDNA linkages at the metaphase-to-anaphase transition. Time-lapse live imaging of the mitotic progression in RPE1 cells expressing GFP-UBF treated with 500 µM dexrazoxane. A cell treated with topoisomerase inhibitor shows impaired sister chromatid segregation and fails to segregate rDNA marked by GFP-UBF. The drug was added shortly before the initiation of imaging. The top panel shows maximum-intensity projections of spinning disk confocal images of eGFP-UBF, and the bottom panel shows maximum-intensity projections of UBF overlaid with the best focal plane phase-contrast images of the cell. The top panel is magnified 1.5× with respect to the bottom panel. Bar, 10 µm. The complete video sequence is shown in Video 3. **(C)** Localization of rDNA and centromeres in cells that divided in the presence of topoisomerase inhibitor dexrazoxane. Asynchronously growing c-Myc–overexpressing RPE1 derivative cell line cMyc-3 was untreated (first left panel) or treated with 500 µM dexrazoxane for 30 min (right four panels). Cells were fixed and labeled by FISH with rDNA probe (green) and CenB probe (red). DNA was counterstained with DAPI. Maximum-intensity projections of confocal images depict cells that failed to segregate the rDNA. Four examples of individual cells are shown (1–4). Arrows point to the rDNA trapped in the cleavage furrow. Bar, 10 µm.

Provided online are three tables in Excel. Tables S1–S3 show a summary of chromosomal spread data from mouse–human hybrid cell line GM15292 and diploid CHON-002 cell line and its tetraploid derivatives, CHON tetraploid 1 and CHON tetraploid 2.

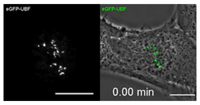

Video 1. **Normal mitosis in an untreated human RPE1 cell stably expressing eGFP-UBF.** UBF is localized to rDNA loci that segregate in anaphase. This video shows the mitotic progression from prophase to telophase. In unperturbed anaphase, no chromatin bridges marked by UBF were observed. Single-plane phase-contrast and fluorescence time-lapse images were obtained every 30 seconds using a spinning disk confocal microscope. The eGFP-UBF panel is magnified 1.5× with respect to overlay. Time is indicated as minutes after initiation of imaging. Bar, 10 µm.

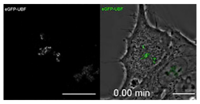

Video 2. **Mitosis in an RPE1 cell stably expressing eGFP-UBF treated with 10 µM topoisomerase inhibitor ICRF-193.** The drug was added shortly before the initiation of imaging. Cells treated with topoisomerase inhibitors show impaired sister chromatid segregation and complete lack of rDNA segregation. Single-plane phase-contrast and fluorescence time-lapse images were obtained every 30 s using a spinning disk confocal microscope. The eGFP-UBF panel is magnified 1.5× with respect to overlay. Time is indicated as minutes after initiation of imaging. Bar, 10 µm.

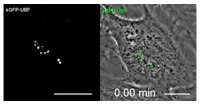

Video 3. **Mitosis in an RPE1 cell stably expressing eGFP-UBF treated with 500 µM topoisomerase inhibitor dexrazoxane.** The drug was added shortly before the initiation of imaging. Cells treated with topoisomerase inhibitors show impaired sister chromatid segregation and complete lack of rDNA segregation. Single-plane phase-contrast and fluorescence time-lapse images were obtained every 30 s using a spinning disk confocal microscope. The eGFP-UBF panel is magnified 1.5× with respect to overlay. Time is indicated as minutes after initiation of imaging. Bar, 10 µm.

References

Ball, G., J. Demmerle, R. Kaufmann, I. Davis, I.M. Dobbie, and L. Schermelleh. 2015. SIMcheck: a Toolbox for Successful Super-resolution Structured Illumination Microscopy. *Sci. Rep.* 5:15915. https://doi.org/10.1038/srep15915

Potapova, T.A., J.R. Unruh, A.C. Box, W.D. Bradford, C.W. Seidel, B.D. Slaughter, S. Sivagnanam, Y. Wu, and R. Li. 2015. Karyotyping human and mouse cells using probes from single-sorted chromosomes and open source software. *Biotechniques*. 59:335–336, 338, 340–342 passim. https://doi.org/10.2144/000114362

Potapova, T.A., C.W. Seidel, A.C. Box, G. Rancati, and R. Li. 2016. Transcriptome analysis of tetraploid cells identifies cyclin D2 as a facilitator of adaptation to genome doubling in the presence of p53. *Mol. Biol. Cell.* 27:3065–3084. https://doi.org/10.1091/mbc.e16-05-0268

Xu, B., H. Li, J.M. Perry, V.P. Singh, J. Unruh, Z. Yu, M. Zakari, W. McDowell, L. Li, and J.L. Gerton. 2017. Ribosomal DNA copy number loss and sequence variation in cancer. *PLoS Genet.* 13:e1006771. https://doi.org/10.1371/journal.pgen.1006771
